# Active catalyst construction for CO_2_ recycling via catalytic synthesis of N-doped carbon on supported Cu

**DOI:** 10.1038/s41467-019-10633-y

**Published:** 2019-06-13

**Authors:** Yajuan Wu, Tao Wang, Hongli Wang, Xinzhi Wang, Xingchao Dai, Feng Shi

**Affiliations:** 1 State Key Laboratory for Oxo Synthesis and Selective Oxidation, Lanzhou Institute of Chemical Physics, Chinese Academy of Sciences, No.18, Tianshui Middle Road, 730000 Lanzhou, China; 2 University of Chinese Academy of Sciences, No. 19A, Yuquan Road, 100049 Beijing, China; 3 SUNCAT Center for Interface Science and Catalysis, Department of Chemical Engineering, Stanford University, Stanford, CA 94305 USA

**Keywords:** Catalyst synthesis, Heterogeneous catalysis, Sustainability

## Abstract

Bridging homogeneous and heterogeneous catalysis is a long-term pursuit in the field of catalysis. Herein, we report our results in integration of nano- and molecular catalysis via catalytic synthesis of nitrogen doped carbon layers on AlOx supported nano-Cu which can finely tune the catalytic performance of the supported copper catalyst. This synthetic catalytic material, which can be generated in situ by the reaction of CuAlOx and 1,10-Phen in the presence of hydrogen, could be used for controllable synthesis of N,N-dimethylformamide (DMF) from dimethylamine and CO_2_/H_2_ via blocking reaction pathways of further catalytic hydrogenation of DMF to N(CH_3_)_3_. Detailed characterizations and DFT calculations reveal that the presence of N-doped layered carbon on the surface of the nano-Cu particles results in higher activation energy barriers during the conversion of DMF to N(CH_3_)_3_. Our primary results could promote merging of homogeneous catalysis and heterogeneous catalysis and CO_2_ recycling.

## Abstract (web summary)

Bridging homogeneous and heterogeneous catalysis is a long-term pursuit in the field of catalysis. Here, the authors present results on integration of nano- and molecular catalysis via catalytic synthesis of nitrogen doped carbon layers on AlO_x_ supported nano-Cu which can finely tune the catalytic performance of the supported copper catalyst.

## Introduction

Utilization and transformation of CO_2_ into value-added chemicals have the potential to alleviate climate change and mitigate the dependence on fossil fuels^1–3^. Among various CO_2_ transformation routes, the reductive functionalization of CO_2_ to DMF is one of the most promising approaches for its chemical utilization, since DMF is a versatile multipurpose reagent in numerous synthetic processes and important solvent having widespread applications in industry^4,5^. Currently, DMF is primarily produced by sodium methoxide catalyzed carbonylation of dimethylamine with carbon monoxide in methanol at 2–10 MPa and 353–373 K^6^. To substitute the toxic carbon monoxide, the reductive coupling of dimethylamine with CO_2_ and H_2_ would offer a fascinating route for sustainable production of DMF. Since the initial report by Haynes et al. on (Ph_3_P)_2_(CO)IrCl catalyzed preparation of DMF from dimethylamine with CO_2_ and H_2_ in 1970^7^, a series of homogeneous and heterogeneous transition-metal catalysts, such as ruthenium^8–11^, palladium^12–14^, platinum^15^, iridium^16^, iron^17,18^, copper^19–22^, and others^23–25^, are developed for the reduction of CO_2_ to synthesize DMF. Although it has been extensively studied, the highly controlled synthesis of DMF remains a tremendous challenge because DMF is prone to further reduction into N(CH_3_)_3_^26^. Therefore, the development of efficient methods for controllable synthesis of DMF from dimethylamine and CO_2_/H_2_ via suppression of further reduction of DMF to N(CH_3_)_3_ is highly desirable, especially by heterogeneous catalysts, because it is easy to be applied in practical process. However, the regulation of the catalytic performance of heterogeneous catalysts on molecular level is difficult. Noteworthy, the development of homogeneous catalysis gives us nice inspirations, in which the catalytic performance can be precisely modulated by organic ligand^27–29^. For example, N-formylation and N-methylation of amines with CO_2_ and phenylsilane can be precisely controlled by organic ligand, i.e., DPPB (1,4-bis(diphenylphosphino)butane) promoted N-methylation whereas Ph_2_CyP (diphenylcyclohexylphosphine) favored for N-formylation^29^. Also, it has been revealed that the addition of molecular organic ligands can tune the catalytic performance of heterogeneous catalysts, too^30–42^. So, a heterogeneous catalyst with the catalytic performance of homogeneous catalyst might be obtained if the traditional organic ligand can be controllably deposited on the surface of heterogeneous catalyst.

**Fig. 1:**
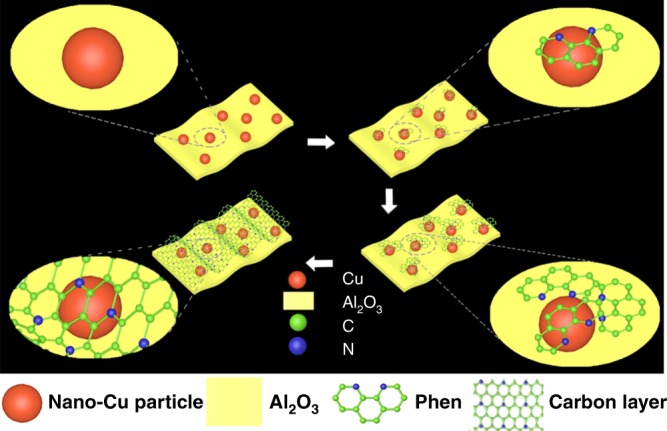
Illustration of carbon coated copper catalyst generation. The nitrogen doped carbon layers can be generated in situ by the reaction of CuAlOx and 1,10-Phen in the presence of hydrogen

It is well known that transition-metal catalysts such as Cu possess the ability of converting the organic molecules into carbon materials, such as CNT (carbon nanotube), graphite/graphene (oxide), amorphous carbon, and ordered mesoporous carbon^43,44^. In addition, Cu was found to be an active catalyst for catalytic hydrogenation of CO_2_ to methanol^45^ and nitrogen-containing ligand was usually used to tuning the catalytic performance of active metals^30,33^. Inspired by the discussions above, a N-doped carbon layer on the surface of nanoparticles might be formed in situ by using the nitrogen-containing ligand as the carbon layer precursor (Fig. 1). In this way, a facile methodology to integrate heterogeneous and homogeneous catalysts can be built, and the preparation of heterogeneous catalyst with homogeneous characteristics for controllable synthesis of DMF with CO_2_/H_2_ may be realized.

Herein, we report a facile methodology for integration of nano- and molecular catalysis via catalytic synthesis of nitrogen doped carbon layers on AlOx supported nano-Cu. This synthetic catalytic material, which can be generated in situ by the reaction of CuAlOx and 1,10-Phen in the presence of hydrogen, could be used for controllable synthesis of DMF from dimethylamine and CO_2_/H_2_ via blocking reaction pathways of further catalytic hydrogenation of DMF to N(CH_3_)_3_.

## Results

### Optimization of the reaction conditions

To validate our hypothesis, we commenced our studies by investigating the reactions of N,N-dimethylammonium N′,N′-dimethylcarbamate (DIMCARB) in the presence of CuAlOx and different organic nitrogen-containing ligands under 3 MPa of CO_2_ and 7 MPa of H_2_ in DME at 160 °C for 24 h (Table 1). The CuAlOx catalyst used here was prepared by a co-precipitation method by adding an aqueous solution of Na_2_CO_3_ to a Cu(NO_3_)_2_ and Al(NO_3_)_3_ solution. It was found that the reaction proceeded smoothly in the absence of ligand leading to DMF and N(CH_3_)_3_ in 93.7% total yield with low selectivity of 41.8% toward DMF (Entry 1). Among the common monodentate nitrogen-containing ligands in this study, the selectivity to DMF slightly decreased when Et_3_N and PhNMe_2_ were used while Py slightly promoted to deliver DMF with 44.0% selectivity (Entries 2–4). Then, various bidentate ligands were also evaluated. Bipy had a negligible impact on the observed selectivity (Entry 5). Clearly, the selectivity toward DMF can be improved slightly if TMEDA was added during the reactions (Entry 6). Compared with TMEDA, the selectivity to DMF sharply increased to 77.9% if DMEDA was employed (Entry 7). In addition, it was discovered that 1,10-Phen exhibited the highest DMF selectivity, i.e., 86.7% (Entry 8). Reducing the amount of 1,10-Phen to 5 mol% resulted in a lower selectivity toward DMF but the same reactivity was kept (Entry 9). Further increasing the amount of 1,10-Phen led to a higher selectivity to DMF (Entries 10–11). Importantly, this catalyst was easily recovered by simple filtration, and it can be reused directly without further treatment. To our delight, 89.6% yield with 97.3% selectivity to DMF was maintained when it was used at the third run, thus, this catalyst exhibits nice reusability (Entry 12).

**Table 1:** Results for DMF synthesis from DIMCARB and CO_2_/H_2_^a^


Entry	Ligand (10 mol%)	Yield (%)^b^	Sel. (%)	
			DMF	N(CH_3_)_3_
1	None	93.7	41.8	58.2
2	Et_3_N	90.0	30.3	69.7
3	PhNMe_2_	89.4	30.3	69.7
4	Py	99.0	44.0	56.0
5	Bipy	91.3	43.6	56.4
6	TMEDA	98.2	52.6	47.4
7	DMEDA	92.1	77.9	22.1
8	1,10-Phen	89.7	86.7	13.3
9^*c*^	1,10-Phen	93.0	78.4	21.6
10^*d*^	1,10-Phen	98.3	92.0	8.0
11^*e*^	1,10-Phen	95.0	97.3	2.7
12^f^	1,10-Phen	89.6	97.3	2.7

^a^DIMCARB (0.5 mmol, equal to 1 mmol HNMe_2_), CuAlOx (100 mg, 9 mmol% Cu), ligand (10 mol%), 1,2-Dimethoxyethane (4 mL), 3 MPa CO_2_, 7 MPa H_2_, 160 °C, 24 h

^b^Combined yield of DMF and N(CH_3_)_3_ were determined by GC-FID using 1,4-dioxane as the internal standard material

^c^1,10-Phen (5 mol%)

^d^1,10-Phen (20 mol%)

^e^1,10-Phen (30 mol%)

^f^The catalyst was reused at the third run. Bipy: 2,2′-bipyridine; Py: pyridine; TMEDA: N,N,N′,N′-Tetramethylethylenediamine; DMEDA: N,N′-dimethylethylenediamine; 1,10-Phen: 1,10-Phenanthroline

### Kinetic and product distribution

**Fig. 2:**
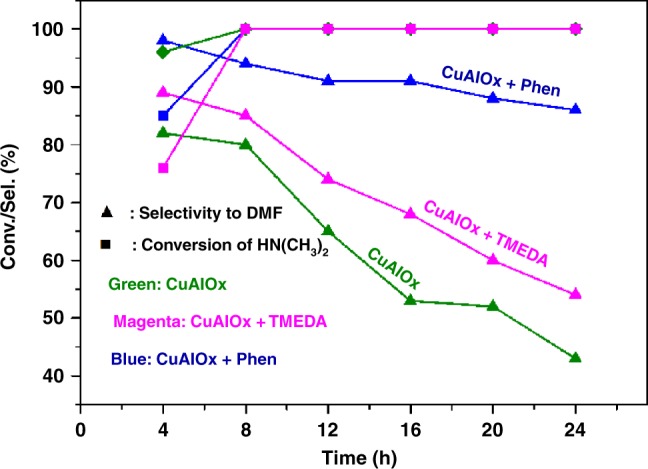
Products distribution during the reactions of DIMCARB with CO_2_ and H_2_. Reaction conditions: DIMCARB (0.5 mmol), CuAlOx (100 mg), ligand (10 mol%), 1,2-Dimethoxyethane (4 mL), 3 MPa CO_2_, 7 MPa H_2_, 160 °C. Except DMF, N(CH_3_)_3_ is the main product

To obtain kinetic and product distribution data, the reactions of DIMCARB with CO_2_ and H_2_ in the presence of CuAlOx, CuAlOx/1,10-Phen, and CuAlOx/TMEDA were traced with varied reaction times. Their performance versus time is shown in Fig. 2. The CuAlOx catalyst or CuAlOx with TMEDA exhibited significant decline in the DMF selectivity with prolonging the reaction time, whereas the high DMF selectivity was still maintained after 24 h if CuAlOx and 1,10-Phen were applied. These results indicated that further reduction of DMF to N(CH_3_)_3_ was suppressed when 1,10-Phen was used as ligand but the activity for DMF generation was not influenced remarkably.

### Catalyst characterization

**Fig. 3:**
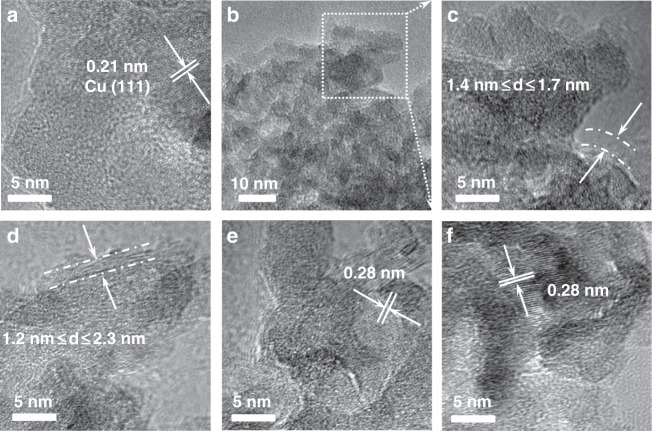
TEM and HRTEM images of the catalysts. HRTEM images of CuAlOx (**a**); CuAlOx + DIMCARB + Phen + CO_2_ + H_2_ (**c**); CuAlOx + Phen + H_2_ (8 h) (**d**); CuAlOx + Phen + H_2_ (16 h) (**e**); CuAlOx + Phen + H_2_ (24 h) (**f**); and TEM image of CuAlOx + DIMCARB + Phen + CO_2_ + H_2_ (**b**). **c** is a magnified picture of a region from **b**. The scale bar of **a**, **c**, **d**, **e**, **f** is 5 nm; **b** is 10 nm

In order to explore whether N-doped carbon layer was formed on the surface of the supported nano-copper and which condition could form a N-doped carbon layer on the surface of nanoparticles, the fresh CuAlOx catalyst and the samples being treated by different conditions were characterized by TEM, XPS, XRD, EXAFS, TGA, and N_2_ adsorption−desorption instruments. At first, the morphology of the CuAlOx samples was examined by TEM and HRTEM as shown in Fig. 3 and Supplementary Fig. 1. The HRTEM image of the fresh CuAlOx provided a lattice fringe with the d-spacing of 0.21 nm, according with the (111) plane of Cu. Compared with fresh catalyst, the HRTEM images of fresh catalyst after being treated with DIMCARB + Phen + CO_2_ + H_2_, Phen + CO_2_ + H_2_, Phen + H_2_ conditions show that the copper nanoparticles are coated with a carbon layer on their surface (shown by an arrow.) The spacing between these layers is around 0.28 nm, which is consistent with that of layered carbon. This result well agrees our hypothesis, i.e., N-doped carbon layer can be in situ synthesized on the heterogenous catalyst sample during the reaction. To trace the formation process of layered carbon, a fresh CuAlOx treated with 1,10-Phen in the presence of H_2_ for 8, 16, and 24 h were also characterized by HRTEM (Fig. 3d–f). Clearly, after being treated for 8 h (Fig. 3d), layered carbon was formed on the edge of catalyst (shown by an arrow). Clearly, the carbon layers grew from edge to surface of CuAlOx after being treated for 16 and 24 h (Fig. 3e, f). The HRTEM images of the fresh CuAlOx catalyst after being treated with Et_3_N, PhNMe_2_, Py, Bipy, TMEDA, and DMEDA revealed that carbon layer was not formed on the surface of CuAlOx catalyst under the same treating conditions.

Furthermore, XPS was performed to explore the chemical state and composition of surface elements in the fresh and treated catalysts (Supplementary Fig. 2). The XPS spectra of all catalysts display two main peaks at 933.5 and 953.5 eV, which are in agreement with Cu 2p_3/2_ and Cu 2p_1/2_ the binding energies of Cu^2+^, respectively. Considering that metallic copper was observed in TEM characterizations, the appearance of Cu^2+^ on catalyst surface should be attributed to the oxidation of Cu(0) when the catalyst samples were exposed to air^19,46,47^. The C 1 s and N 1 s spectra of the catalysts were also analyzed by XPS (Supplementary Fig. 3). However, the C1s spectra were overlapped by the organic carbon from environment, and the N1s spectra were too weak due to the low concentration. So the structure of the carbon layers can not be determined by XPS analysis.

According to the nitrogen adsorption–desorption isotherms, the formation of mesoporous structure for the fresh CuAlOx catalyst was observed (Supplementary Fig. 4). Clearly, it was composed of pores with diameters of 5.1 and 12.2 nm. It should be noted that the porous structure partly disappeared after the treatment of fresh catalyst under different conditions, which should be attributed to that formation of layered carbon, resulting in clogged mesoporous of the fresh CuAlOx catalyst. The N_2_ adsorption−desorption tests showed that the BET surface areas of the fresh and treated catalysts (Supplementary Table 1) were 250.3−330.3 m^2^ g^−1^.

**Fig. 4:**
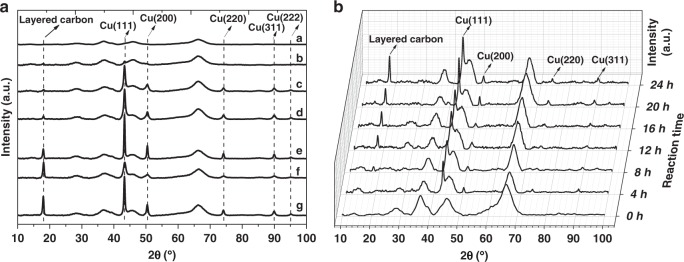
XRD patterns of the samples. **a** XRD patterns of CuAlOx catalysts treated in different conditions: CuAlOx (curve a); CuAlOx + DIMCARB + CO_2_ + H_2_ (curve b); CuAlOx + Phen + CO_2_ (curve c); CuAlOx + Phen + Ar (curve d); CuAlOx + DIMCARB + Phen + CO_2_ + H_2_ (curve e); CuAlOx + Phen + CO_2_ + H_2_ (curve f); and CuAlOx + Phen + H_2_ (curve g). **b** Time-resolved XRD patterns of CuAlOx catalysts treated with 1,10-Phen and H_2_ at different times: 0, 4, 8, 12, 16, 20, and 24 h

Then, fresh CuAlOx catalyst and the samples being treated by different conditions were characterized by XRD measurement, too (Fig. 4a and Supplementary Fig. 5). The fresh CuAlOx samples show diffraction peaks at 25.3, 37.9, 48.2, 54.1, and 55.3, which can be assigned as (101), (004), (200), (105), and (211) reflection lines of γ-Al_2_O_3_. The treated catalysts showed XRD reflections at 43.3°, 50.4°, 74.1°, 89.9°, and 95.0°, which are ascribed to the metallic copper phase. As the copper particle size can not be precisely determined by TEM, in which the copper particles were overlapped with AlOx, the crystallite sizes of different catalysts were calculated from Cu (111) diffraction peak in XRD by using Scherrer equation. As shown in Supplementary Table 1, these samples have a copper crystallite size in the range from 5.1 to 19.9 nm. Besides, the fresh CuAlOx catalyst treated with DIMCARB + Phen + CO_2_ + H_2_, Phen + CO_2_ + H_2_, Phen + H_2_ conditions exhibited a new diffraction peak located at 18.0°. This peak is the typical diffraction peak of N-dope carbon (PDF#51–2183), which clearly proves our hypothesis. In addition, no diffraction peak was observable at 18.1° in fresh catalyst after being treated with Et_3_N, PhNMe_2_, Py, Bipy, TMEDA, and DMEDA. These results revealed that layered carbon was not formed on the surface of CuAlOx catalyst under the above conditions, which is in line with the TEM results. Therefore, we could draw conclusion that other ligands are not able to modulate the selectivity because of lack formation of the carbon shell on the catalysts.

Following, time-resolved XRD patterns of CuAlOx catalysts treated with 1,10-Phen and H_2_ were collected in order to trace the formation progress of carbon layer (Fig. 4b). Clearly, a new peak at 18.0° appears and slowly enhancing, representing the layered carbon. This unambiguously confirmed that layered N-doped carbon coated on the surface of nanoparticle can be synthesized by reaction of CuAlOx and 1,10-Phen in the presence of H_2_. TG analysis (Supplementary Fig. 6) was used to gain the quantity of carbon layers or adsorbed organic molecules on CuAlOx after being treated under different conditions, which were 2.5–12.0 wt% (Supplementary Table 2). The corresponding copper loadings were 3.67–5.58 wt%.

**Fig. 5:**
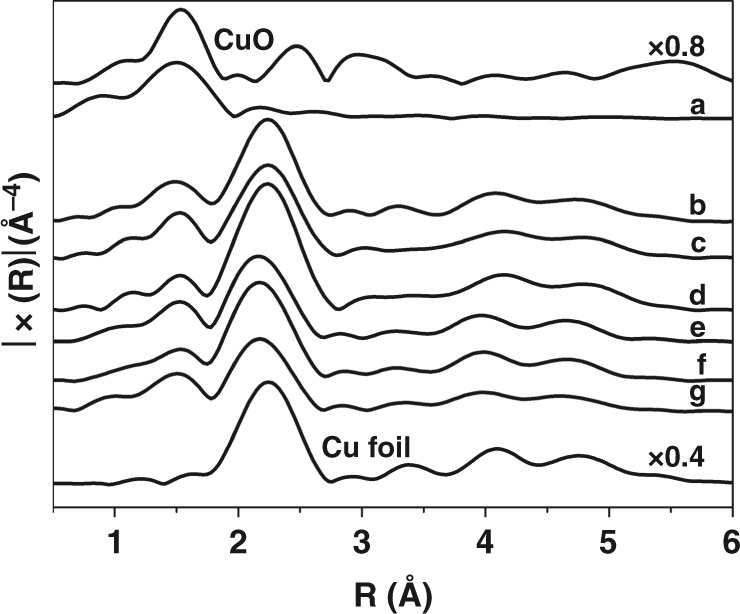
Fourier transform (FT) of Cu K-edge EXAFS. CuAlO_x_ (curve a); CuAlO_x_ + DIMCARB + CO_2_ + H_2_ (curve b); CuAlO_x_ + Phen + CO_2_ (curve c); CuAlO_x_ + Phen + Ar (curve d); CuAlO_x_ + DIMCARB + Phen + CO_2_ + H_2_ (curve e); CuAlO_x_ + Phen + CO_2_ + H_2_ (curve f); CuAlO_x_ + Phen + H_2_ (curve g); and Cu foil

Next, the Fourier transformed Cu K-edge EXAFS spectra were analyzed to reveal the coordination environment of Cu in CuAlOx catalyst samples (Fig. 5). The fitting results including coordination number, bond distance were listed in Supplementary Table 3. The Cu K-edge for fresh CuAlOx catalyst shows some resemblance to the CuOx reference, which may be due to the oxidation of Cu(0) when exposed to air and average particle size remains small. The results from fitting the Cu K edge EXAFS spectra show that the bond distance of Cu−Cu remains constant and a slight increase in the Cu−Cu coordination number (0.5–6.9) after CuAlOx catalyst being treated, indicating Cu particle size slightly increases after treatment. This result confirms the observations from XRD patterns.

**Fig. 6:**
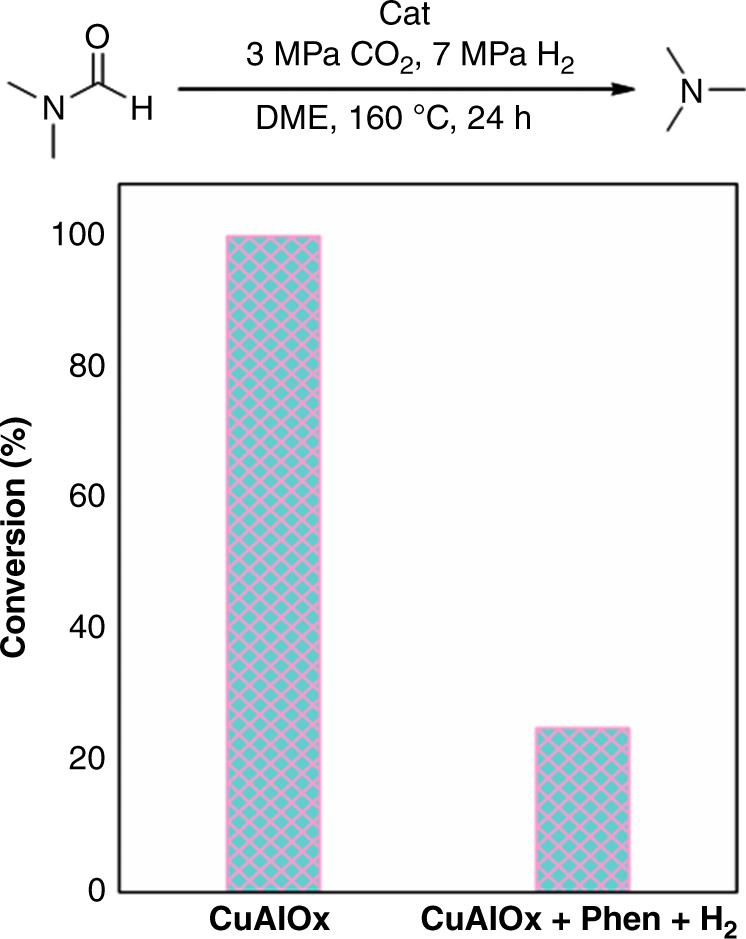
Catalytic hydrogenation of DMF with different catalysts. Hundred percent conversion of DMF to N(CH_3_)_3_ was observed with CuAlOx as catalyst while it was only 25% conversion if CuAlOx treated by 1,10-Phen and H_2_ was applied as catalyst

To verify whether this structure have the ability as we hypothesized, control experiments were conducted under the same conditions. In the reaction of DIMCARB with CO_2_ and H_2_, fresh CuAlOx catalyst produced DMF and N(CH_3_)_3_ in 93.7% combined yield with low DMF selectivity of 41.8%. If applying N-doped layer carbon coated nano-copper (CuAlOx catalyst treated with 1,10-Phen and H_2_) as the catalyst, 95.8% combined yield with much higher DMF selectivity of 80.2% was obtained. In addition, for the catalytic hydrogenation of DMF, fresh CuAlOx catalyst afforded 100% conversion while CuAlOx treated with 1,10-Phen and H_2_ only gave 25% conversion (Fig. 6). The results obviously demonstrated that the precisely synthesized catalyst indeed possesses ability for controllable synthesis of DMF from CO_2_/H_2_. The copper contents in the solution after each cycle were tested by ICP-AES (Supplementary Table 4). 2.970, 1.928, and 0.900 ppm copper were observed in the solutions with CuAlOx itself as catalyst at the first, second, and third runs, while it was only 0.513, 0.028, and 0.013 ppm if Phen was added. Therefore, except the improving of the catalytic performance for DMF synthesis, the formation of carbon layer also can stabilize the CuAlOx catalyst.

### DFT calculations

**Fig. 7:**
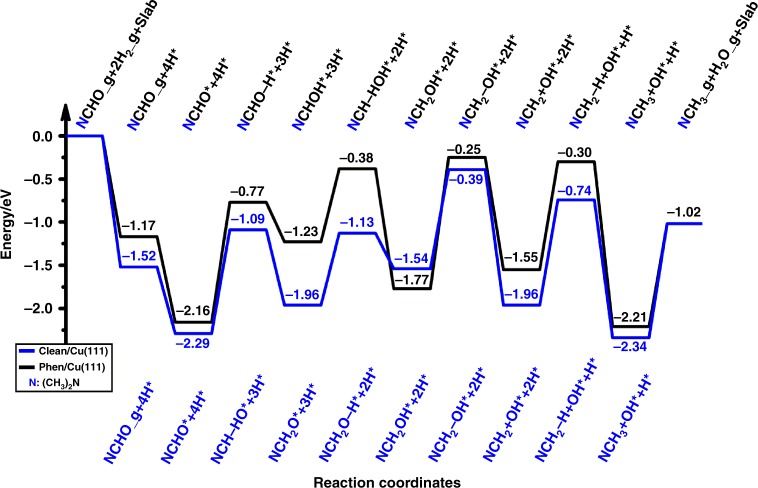
Potential energy diagram for the most favorable reaction pathway. black line: DMF hydrogenation to N(CH_3_)_3_ on clean Cu(111) surfaces; blue line: DMF hydrogenation to N(CH_3_)_3_ on Phen-covered Cu(111) surfaces

In order to explore the role of N-doped nano-carbon layer in controlling the selective synthesis of DMF, DFT calculations were performed to simulate the detailed reaction mechanism of DMF hydrogenation to N(CH_3_)_3_ on clean and Phen-covered Cu(111) surfaces, where five reaction pathways are considered as shown in Supplementary Figs. 8 and 9. More specifically, the adsorbed (CH_3_)_2_NCHO could produce (CH_3_)_2_NCH + O species via C–O bond breaking, and further hydrogenation of (CH_3_)_2_NCH would produce undesired N(CH_3_)_3_ species, which finally results in the lose in (CH_3_)_2_NCHO selectivity. (CH_3_)_2_NCHO could also generate (CH_3_)_2_NCH_2_O and (CH_3_)_2_NCHOH species via hydrogenation. Then, (CH_3_)_2_NCH_2_O could produce either (CH_3_)_2_NCH_2_ + O species via C–O bond breaking or (CH_3_)_2_NCH_2_OH species via deep hydrogenation. Similarly, (CH_3_)_2_NCHOH could produce either (CH_3_)_2_NCH + OH species via C–O bond breaking or (CH_3_)_2_NCH_2_OH species via hydrogenation. Furthermore, (CH_3_)_2_NCH_2_OH could produce (CH_3_)_2_NCH_2_ + OH species via C–O bond breaking. Finally, the (CH_3_)_2_NCH and (CH_3_)_2_NCH_2_ species will be hydrogenated to N(CH_3_)_3_ species. All the detailed energetics as well as structures of all the species and transition states could be found in Supplementary Figs. 10 to 13. On the basis of the systematic reaction mechanism computations, we are able to have direct comparison the difference between clean and Phen-covered Cu catalyst. Figure 7 depicts the potential energy diagrams for the most favorable reaction pathways of DMF hydrogenation to N(CH_3_)_3_ on clean and Phen-covered Cu(111) surfaces. Clearly, the presence of Phen on Cu(111) surface increases the energy barriers of C–H bond formation and C-O bond breaking in DMF hydrogenation, which indicates its lower DMF hydrogenation activity compared with clean Cu(111) surface. Therefore, the computational results can reasonably explain our experimental findings.

In conclusion, we have successfully developed a facile methodology to integrate heterogeneous and homogeneous catalysts via construction of N-doped carbon layer on the surface of supported nano-catalyst. The synthetic catalytic material with defined structure exhibited a remarkable catalytic performance in contrallable synthesis of DMF. Also, the applying of this concept in other catalytic hydrogenation reactions were ongoing. This work offers an effective methodology for the precise and controlled synthesis of heterogeneous catalysts with defined active sites, and it may provide an important insight in merging homogeneous catalysis with heterogeneous catalysis as well as controlled catalytic conversion of CO_2_.

## Methods

### Preparation of CuAlOx

0.145 g (0.6 mmol) of Cu(NO_3_)_2_·3H_2_O and 4.275 g (11.4 mmol) of Al(NO_3_)_3_·9H_2_O were added into 125 mL deionized water at room temperature in a 250 mL flask. Then, 38 mL of Na_2_CO_3_ solution (0.93 mol/L) was added dropwise into the solution under vigorous stirring and the mixture was stirred for a further 5 h at room temperature. The reaction mixture was centrifuged and washed with water to remove the base until the pH value of the aqueous solution was ≈7. Subsequently, the solid was dried at 100 °C overnight, calcined at 350 °C for 12 h, and then reduced under hydrogen flow at 350 °C for 3 h. Finally, a black powder was obtained and denoted as CuAlO_x_.

### N-formylation of dimethylamine with CO_2_/H_2_

A mixture of DIMCARB (67 mg), CuAlOx (100 mg), 1,10-Phen (0–0.15 mmol), and 1,2-dimethoxyethane (4 mL) were added in a 100 mL autoclave. The autoclave was sealed and exchanged with CO_2_ three times and reacted at 160 °C (oven temperature 180 °C) under 3 MPa CO_2_ and 7 MPa H_2_ for 24 h. After cooling to room temperature, the autoclave was placed in a cold trap (−20 °C) for 20 min and the gas was pumped slowly into 120 mL 2 mol/L HCl methanol solution to absorb the gaseous N(CH_3_)_3_. Then, the methanol solution was concentrated under vacuum and the yield of gaseous N(CH_3_)_3_ was obtained. Subsequently, 0.5 mL triethylamine and 15 mL 1,2-dimethoxyethane were added into the reaction mixture quickly. The GC yield of liquid N(CH_3_)_3_ and DMF were determined by GC-FID (Agilent 7890 A) using 1,4-dioxane as the internal standard. The overall yield of N(CH_3_)_3_ was calculated by adding gaseous and liquid yields together.

### Procedure for recycling test

The used catalyst was separated from the reaction mixtures by centrifuging, washed three times alternately with DME and acetone. After being dried in air at room temperature, it was recovered and directly recharged into the reaction tube for the next run.

### The procedures for CuAlOx catalyst treated

For CuAlO_x_ + DIMCARB + Phen + CO_2_ + H_2_, the processing condions are the same as that of N-formylation of dimethylamine. After cooling to room temperature, the catalyst was washed alternately with methanol and acetone for three times and then dried in the air. For CuAlO_x_ + DIMCARB + CO_2_ + H_2_ and CuAlO_x_ + Phen + CO_2_ + H_2_, the processing conditions are the same with the reaction conditions without adding Phen or DIMCARB. For CuAlO_x_ + Phen + CO_2_, CuAlO_x_ + Phen + H_2_ and CuAlO_x_ + Phen + Ar, the processing conditions are consistent with the reaction conditions with only 3 MPa of gaseous atmosphere (CO_2_/H_2_/Ar).

### DFT calculations

All calculations were performed by using the plane-wave based DFT method as implemented in the Vienna ab initio simulation package (VASP)^48,49^. Periodic slab models were used to model the Cu catalyst. The electron ion interaction was described with the projector augmented wave (PAW) method^50,51^. The electron exchange and correlation energy was treated within the generalized gradient approximation in the Perdew-Burke-Ernzerhof formalism (GGA-PBE)^52^. The density-dependent dDsC method was used for the dispersion correction^53^. More computational details could be found in Supplementary Material.

## Supplementary information

Supplementary Information

## Data Availability

All data are available from the corresponding authors upon reasonable request.
