# Supplementary material for: Active catalyst construction for CO2 recycling via catalytic synthesis of N-doped carbon on supported Cu
Source: Nat Commun. 2019 Jun 13;10:2599. doi: 10.1038/s41467-019-10633-y (PMC6565717; doi:10.1038/s41467-019-10633-y)
Supplement: Supplementary file 1 — Supplementary Information [file 41467_2019_10633_MOESM1_ESM.pdf]

Supplementary Information

Active catalyst construction for CO_2 recycling via catalytic synthesis of N-doped carbon on supported Cu

Wu et.al

Supplementary Methods

Materials: All solvents and chemicals were obtained commercially and were used as received.

Characterization Methods: The contents of Cu in the catalysts were measured by inductively coupled plasma-atomic emission spectrometry (ICP-AES), using an Iris advantage Thermo Jarrel Ash device. XRD measurements were conducted by a STADIP automated transmission diffractometer (STOE) equipped with an incident beam curved germanium monochromator selecting Cu Kα1 radiation and a 6 ° position sensitive detector (PSD). The XRD patterns are scanned in the 2θ range of 10-100 °. For the data interpretation the software WinXpow (STOE) and the database of Powder Diffraction File (PDF) of the International Centre of Diffraction Data (ICDD) were used. XPS were obtained using a VG ES-CALAB 210 instrument equipped with a dual Mg/Al anode X-ray source, a hemispherical capacitor analyzer, and a 5 keV Ar^+ iron gun. The electron binding energy was referenced to the C 1s peak at 284.8 eV. The background pressure in the chamber was less than 10^{-7} Pa. The peaks were fitted by Gaussian–Lorentzian curves after a Linear background subtraction. For quantitative analysis, the peak area was divided by the element-specific Scofield factor and the transmission function of the analyzer. TEM analysis was carried out on a FEI G2F20 transmission electron microscope operating at 200 KeV. Nitrogen adsorption-desorption isotherms were measured at 77 K using American Quantachrome iQ_2 automated gas sorption analyzer. The pore-size distribution was calculated by Barrett, Joyner and Halenda (BJH) method from desorption isotherm. Thermogravimetric analysis (TGA) was performed on a METTLER TOLEDO simultaneous thermal analyzer in an air atmosphere from 30 °C to 350 °C and then maintained at 350 °C for 1 h. Extended X-ray absorption fine structure (EXAFS) experiments were performed at the Beijing Synchrotron Radiation Facility (BSRF) in Institute of High Energy Physics, Chinese Academy of Sciences with storage ring energy of 2.5 GeV and a beam current between 150 and 250 mA. The Cu K edge absorbance of powder catalysts was measured in transmission geometry at room temperature. The energy was scanned from -200 eV below to 800 eV above the Cu K edge (8979 eV). EXAFS data analysis was carried out using ifeffit analysis programs (http://cars9.uchicago.edu/ifeffit/).

Computational Calculations Methods: All calculations were performed by using the plane-wave based DFT method as implemented in the Vienna ab initio simulation package (VASP)[1,2]. Periodic slab models were used to model the Cu catalyst. The electron ion interaction was described with the projector augmented wave (PAW) method[3,4]. The electron exchange and correlation energy was treated within the generalized gradient approximation in the Perdew-Burke-Ernzerhof formalism (GGA-PBE)[5]. To acquire accurate energies with errors less than 1 meV per atom, systematic convergence test was performed for the choice of cutoff energies. As listed in **Supplementary Table 5**, we could acquire accurate energies as long as cutoff energy is higher than 330 eV for Cu catalyst. To guarantee enough accuracy, we finally chose an cutoff energy of 400 eV for all computations. A second-order Methfessel-Paxton[6] electron smearing with $\sigma = 0.2$ eV were used. A vacuum layer of 15 Å was set between the periodically repeated slabs to avoid strong interactions. The density-dependent dDsC method was used for the dispersion correction[7]. All the structure optimizations were done when the force tolerance and energy difference became lower than 0.02 eV/Å and 10–5 eV, respectively.

Calculation Models: Calculation of the fcc-bulk Cu crystal structure with a k-point mesh of 9×9×9 gives a lattice constant of 3.62 Å. To elucidate the role of phen in determining the catalytic performance of Cu catalyst in DFM hydrogenation to $N(CH_3)_3$ reaction, two models were applied (model **A**: clean Cu(111) surface and model **B**: Phen-covered Cu(111) surface). To have a reasonable model for our mechanism simulations, the thickness of Cu(111) surface was tested, where the adsorption energy of $(CH_3)_2NCHO$ was used as the criteria for the test. As shown in **Supplementary Table 6**, the thickness did not show obious effects on $(CH_3)_2NCHO$ adsorption energies. To balance the computationl accuracy and cost, we finally chose a *p*(3×3)-3L and *p*(4×5)-3L supercells to simulate the clean and Phen covered Cu(111) surfaces, respectively. In all these models, the first layer was fully relaxed, while the bottom two layers were fixed as the bulk. In addition, the choice of k-point mesh for sampling the Brillouin zone was also tested, and adsorption energy of $(CH_3)_2NCHO$ was still used as the indicator. As shown in **Supplementary Table 7**, a 3×3×1 k-point mesh is good enough to get converged $(CH_3)_2NCHO$ adsorption energies on clean Cu(111) surface, while a 3×2×1 k-point is fine for the Phen-covered Cu(111) surface. The schematic structures of the models are shown in **Supplementary Figure 7**.

To identify the most stable adsorption configurations for each intermediate in the reaction mechanism, we put each species on different sites on Cu(111) surface, which has four possible adsorption sites (top, bridge, fcc and hcp hollow). Then structure optimizations were performed with DFT method. Finally, we applied the most stable one as the initial state for further transition state calculations. The adsorption energies of each species was defined as $E_{ads} = E(\text{adsorbate/surface}) - E(\text{surface}) - E(\text{adsorbate})$, where E(adsorbate/surface), E(surface), and E(adsorbate) are the total energies of species adsorbed on the surface, clean surface model and species in the gas phase, respectively. Transition-state structures are estimated using the climbing image nudged elastic band method (CI-NEB)[8]. That is, seven images were interpolated between initial and final state for a given elementary step. The energy barrier (E_a) is calculated according to $E_a = E_{TS} - E_{IS}$, and the reaction energy is defined as $E_r = E_{FS} - E_{IS}$, where E_{IS}, E_{FS} and E_{TS} are the total energies of the initial state (IS), final state (FS) and transition state (TS) of each elementary step. All transition states are verified by vibration analyses with only one imaginary frequency, and all the imaginary frequencies of each transition states are given in **Supplementary Figures 8 and 13**. To build the potential energy diagrams for the reaction mechanisms, all the energies are referred to the total energy of gaseous $(CH_3)_2CHO$ + gaseous H_2 + slab.

Data descriptions: To simulate the selectivity difference between pure and Phen covered Cu catalyst, we performed detailed reaction mechanism computations with DFT from DMF to $N(CH_3)_3$. We propose that this reaction is a key channel in determining the selectivity. As shown in **Supplementary Figure 8**, five reaction pathways from $(CH_3)_2NCHO$ to $N(CH_3)_3$ are considered on clean Cu(111) surface. For example, $(CH_3)_2NCHO$ could generate $(CH_3)_2NCH$ + O species via C-O bond breaking, and further hydrogenation of $(CH_3)_2NCH$ would produce undesired $N(CH_3)_3$ species, which finally affect the selective production of $(CH_3)_2NCHO$. $(CH_3)_2NCHO$ could also generate $(CH_3)_2NCH_2O$ and $(CH_3)_2NCHOH$ species via hydrogenation. Then, $(CH_3)_2NCH_2O$ could produce either $(CH_3)_2NCH_2$ + O species via C-O bond breaking or $(CH_3)_2NCH_2OH$ species via hydrogenation. Similarly, $(CH_3)_2NCHOH$ could produce either $(CH_3)_2NCH$ + OH species via C-O bond breaking or $(CH_3)_2NCH_2OH$ species via hydrogenation. Furthermore, $(CH_3)_2NCH_2OH$ could produce $(CH_3)_2NCH_2$+OH species via C-O bond breaking. Finally, the $(CH_3)_2NCH$ and $(CH_3)_2NCH_2$ species will be hydrogenated to $N(CH_3)_3$ species. All the detailed

energetics as well as structures of all the species and transition states could be found in **Supplementary Figures 9 and 10**. The same reaction mechanisms were also calculated on the Phen-covered Cu(111) surfaces, and all the energetics and structural information are shown in **Supplementary Figures 11-13**.

Supplementary Tables

Supplementary Table 1 | The physical properties of the samples

Entry	Catalyst	Wt_{Cu} (%)[a]	S_{BET} (m^2/g)[b]	V_p (m^3/g)[b]	D_p (Å)[b]	d_{Cu} (nm)[c]
1	CuAlOx	5.86	315.73	0.77	4.90	5.07
2	CuAlOx+ DIMCARB +Phen+CO_2+H_2	5.35	279.61	0.63	4.31	19.9
3	CuAlOx+DIMCARB+CO_2+H_2	4.45	250.30	0.73	21.53	6.66
4	CuAlOx +Phen+CO_2+H_2	5.32	286.86	0.59	4.89	7.94
5	CuAlOx +Phen+CO_2	5.25	265.77	0.58	24.47	12.90
6	CuAlOx +Phen+H_2	4.22	330.34	0.59	4.89	16.70
7	CuAlOx +Phen+Ar	5.58	264.77	0.55	24.49	12.50
8	CuAlOx+DIMCARB+Et_3N+CO_2+H_2	4.07	277.28	0.41	4.32	8.93
9	CuAlOx+DIMCARB+$PhNMe_2$+CO_2+H_2	4.01	250.96	0.42	21.55	8.27
10	CuAlOx+DIMCARB+Py+CO_2+H_2	3.93	313.69	0.47	4.31	10.20
11	CuAlOx+DIMCARB+Bipy+CO_2+H_2	3.87	281.89	0.47	21.48	9.70
12	CuAlOx+DIMCARB+TMEDA+CO_2+H_2	3.67	308.28	0.46	4.90	7.34
13	CuAlOx+DIMCARB+DMEDA+CO_2+H_2	3.75	333.01	0.62	19.15	9.03

[a]Determined by ICP-AES. [b]Determined by an IQ_2 automated gas sorption analyser. S_{BET}:Surface area; V_p: Pore volume; D_p: Average pore diameter. [c]Particle size calculated from Cu(111) diffraction peak in XRD by using Scherrer equation.

Supplementary Table 2 | The contents of N doped layered carbon loadings/absorbed organic molecules and copper

Entry	Catalyst	Layered carbon loadings/absorbed organic molecules (%)[a]	Wt_{Cu} (%)[b]
1	$CuAlO_x$	--	5.86
2	$CuAlO_x$+Phen+H_2-8 h	2.5	--
3	$CuAlO_x$+Phen+H_2-16 h	3.4	--
4	$CuAlO_x$+Phen+H_2-24 h	6.0	4.22
5	$CuAlO_x$+Phen+Ar	6.6	5.58
6	$CuAlO_x$+Phen+CO_2;	6.9	5.25
7	$CuAlO_x$+DIMCARB+TMEDA+CO_2+H_2	8.0	3.67
8	$CuAlO_x$+DIMCARB+Py+CO_2+H_2	8.3	3.93
9	$CuAlO_x$+Phen+CO_2+H_2	8.4	5.32
10	$CuAlO_x$+DIMCARB+Bipy+CO_2+H_2	8.9	3.87
11	$CuAlO_x$+DIMCARB+Phen+CO_2+H_2	9.7	5.35
12	$CuAlO_x$+DIMCARB+$PhNMe_2$+CO_2+H_2	11.1	4.01
13	$CuAlO_x$+DIMCARB+Et_3N+CO_2+H_2	11.4	4.07
14	$CuAlO_x$+DIMCARB+CO_2+H_2	11.4	4.45
15	$CuAlO_x$+DIMCARB+DMEDA+CO_2+H_2	12.0	3.75

[a]Calculated from the TG percentage of CuAlOx at 5520 s minus the TG percentage of any a sample at 5520 s (The samples were heated from 30 °C to 350 °C in the first 1920 s and then maintained at 350 °C from 1920 s to 5520 s) ; [b]Determined by ICP-AES.

Supplementary Table 3 | Quantitative analyses of Cu-Cu and Cu-O contributions as measured by EXAFS

Samples	Shell	CN[a]	R(Å)[b]	σ^2 (Å^2)	ΔE_0 (eV)	R factor
Cu foil	Cu-Cu	12	2.54	0.008	3.75	0.00256
CuO	Cu-O	4	1.95	0.004	-0.83	0.00770
CuAlOx	Cu-Cu	0.5	2.67	0.010	9.79	0.0026
	Cu-O	3.9	1.95	0.006	-2.84	
CuAlOx+DIMCARB+CO_2+H_2	Cu-Cu	5.5	2.54	0.009	3.13	0.00167
	Cu-O	1.6	1.90	0.005	6.21	
CuAlOx+Phen+CO_2+H_2	Cu-Cu	4.2	2.55	0.009	-6.43	0.00483
	Cu-O	2.3	1.91	0.005	-5.24	
CuAlOx+Phen+Ar	Cu-Cu	6.9	2.54	0.009	3.80	0.00044
	Cu-O	1.1	1.90	0.005	7.70	
CuAlOx+DIMCARB+Phen+CO_2+H_2	Cu-Cu	4.9	2.54	0.009	-8.33	0.00112
	Cu-O	1.6	1.93	0.004	-1.73	
CuAlOx+Phen+CO_2+H_2	Cu-Cu	4.2	2.55	0.009	-6.43	0.00483
	Cu-O	2.3	1.91	0.005	-5.24	
CuAlOx+Phen+H_2	Cu-Cu	5.9	2.54	0.009	-6.79	0.00665
	Cu-O	1.4	1.92	0.005	-1.40	

Data from Extended Data Supplementary Figure 7 [a]**CN,** coordination number. [b]**R**, distance between absorber and backscattered atoms.

Supplementary Table 4 | Cu contents of the recycled catalysts after each cycle[a]

Entry	times	Fresh catalyst	Fresh catalyst treated with 1,10-Phen
		Cu (ppm/solution)	Cu (ppm/solution)
1	1st	2.970	0.513
2	2rd	1.928	0.028
3	3th	0.900	0.013

[a] Determined by ICP-AES.

Supplementary Table 5 | The convergence test of cutoff energies for Cu bulk

Cutoff Energy (eV)	bulk Cu energy (eV)	Error (eV / atom)
300	-3.72725	
310	-3.73096	0.004
320	-3.73297	0.002
330	-3.73386	0.001
340	-3.73385	0.000
350	-3.73316	0.001
360	-3.73215	0.001
370	-3.73101	0.001
380	-3.72985	0.001
390	-3.72879	0.001
400	-3.72803	0.001
410	-3.72742	0.001
420	-3.72671	0.000
430	-3.72662	0.000
440	-3.72651	0.000
450	-3.72663	0.000

Supplementary Table 6 | The adsorption energies of $(CH_3)_2NCHO$ on Cu(111) surface with different atomic layers

Model thickness (relaxed / fixed)	E_{ads} (NCHO) / eV
Cu(111)-2L(1/1)	-0.79
Cu(111)-3L (1/2)	-0.77
Cu(111)-4L (1/3)	-0.78
Cu(111)-5L (1/4)	-0.79

Supplementary Table 7 | The adsorption energies of $(CH_3)_2NCHO$ on Cu(111) and Phen-covered Cu(111) surfaces with different k-point meshes.

**Clean Cu(111)**		**Phen-covered Cu(111)**	
KPOINTS	E_{ads} (NCHO) / eV	KPOINTS	E_{ads} (NCHO) / eV
(1×1×1)	-1.12	(1×1×1)	-0.86
(2×2×1)	-0.86	(2×1×1)	-0.88
(3×3×1)	-0.77	(2×2×1)	-0.99
(4×4×1)	-0.77	(3×2×1)	-1.00
(5×5×1)	-0.76	(3×3×1)	-1.01

Supplementary Figures

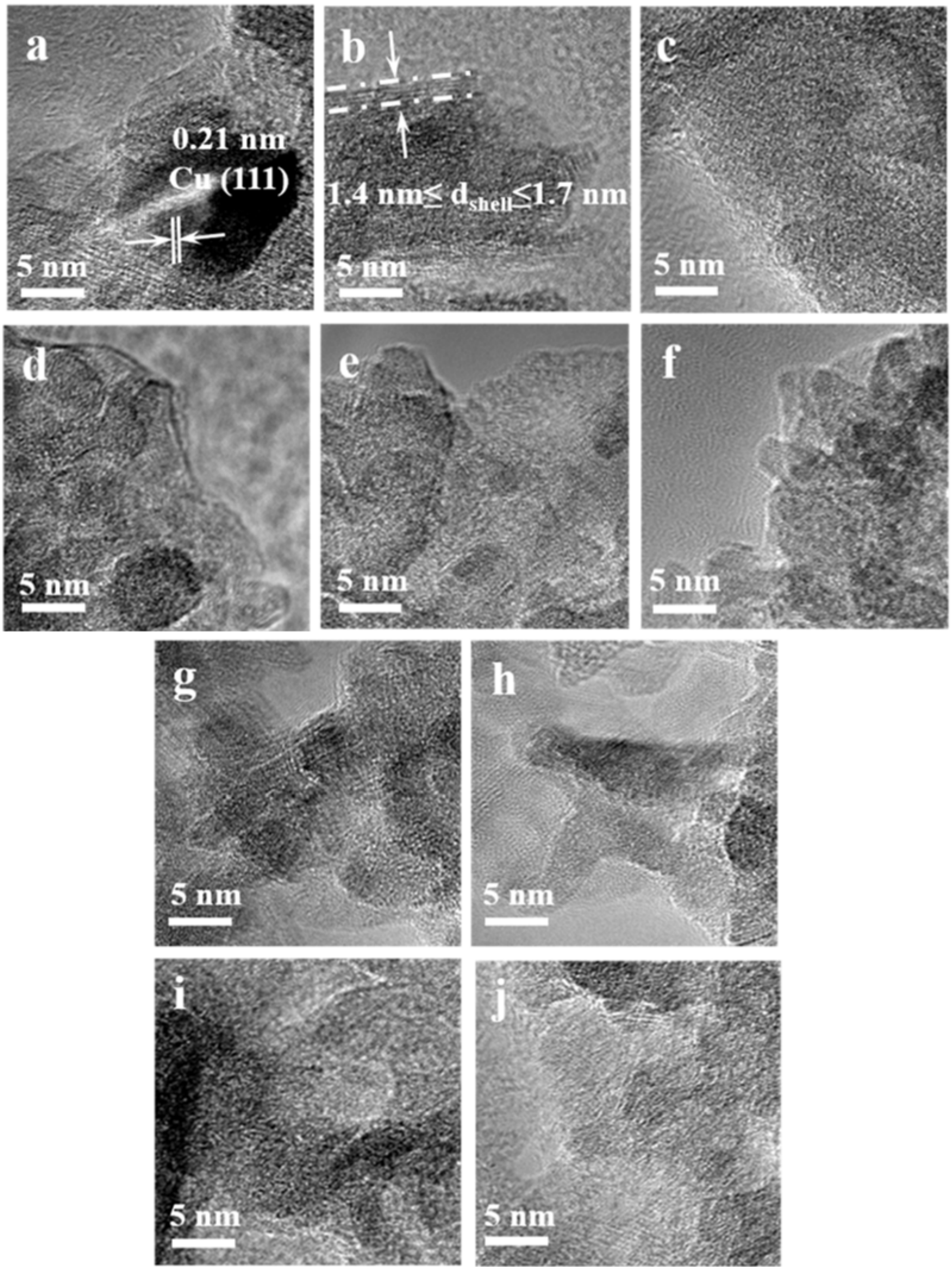

Supplementary Figure 1 | HRTEM images of the samples. **a,** $CuAlO_x$+DIMCARB+CO_2+H_2; **b,** $CuAlO_x$+Phen+H_2; **c,** $CuAlO_x$+Phen+CO_2; **d,** $CuAlO_x$+Phen+Ar; **e,** $CuAlO_x$+DIMCARB+Et_3N+CO_2+H_2; **f,** $CuAlO_x$+DIMCARB+$PhNMe_2$+CO_2+H_2; **g,** $CuAlO_x$+DIMCARB+Py+CO_2+H_2; **h,** $CuAlO_x$+DIMCARB+Bipy+CO_2+H_2; **i,** $CuAlO_x$+DIMCARB+TMEDA+CO_2+H_2; **j,** $CuAlO_x$+DIMCARB+DMEDA+CO_2+H_2.

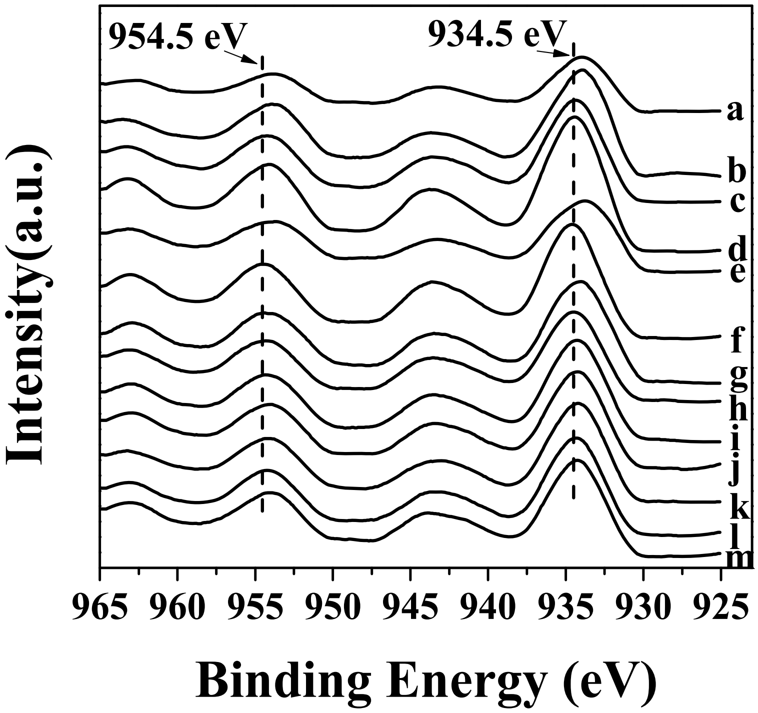

Supplementary Figure 2 | Cu2p XPS spectra of the catalysts. **a,** $CuAlO_x$; **b,** $CuAlO_x$+DIMCARB+Phen+CO_2+H_2; **c,** $CuAlO_x$+DIMCARB+CO_2+H_2; **d,** $CuAlO_x$+Phen+CO_2+H_2; **e,** $CuAlO_x$+Phen+CO_2; **f,** $CuAlO_x$+Phen+H_2; **g,** $CuAlO_x$+Phen+Ar; **h,** $CuAlO_x$+DIMCARB+Et_3N+CO_2+H_2; **f,** $CuAlO_x$+DIMCARB+$PhNMe_2$+CO_2+H_2; **g,** $CuAlO_x$+DIMCARB+Py+CO_2+H_2; **h,** $CuAlO_x$+DIMCARB+Bipy+CO_2+H_2; **i,** $CuAlO_x$+DIMCARB+TMEDA+CO_2+H_2; **j,** $CuAlO_x$+DIMCARB+DMEDA+CO_2+H_2.

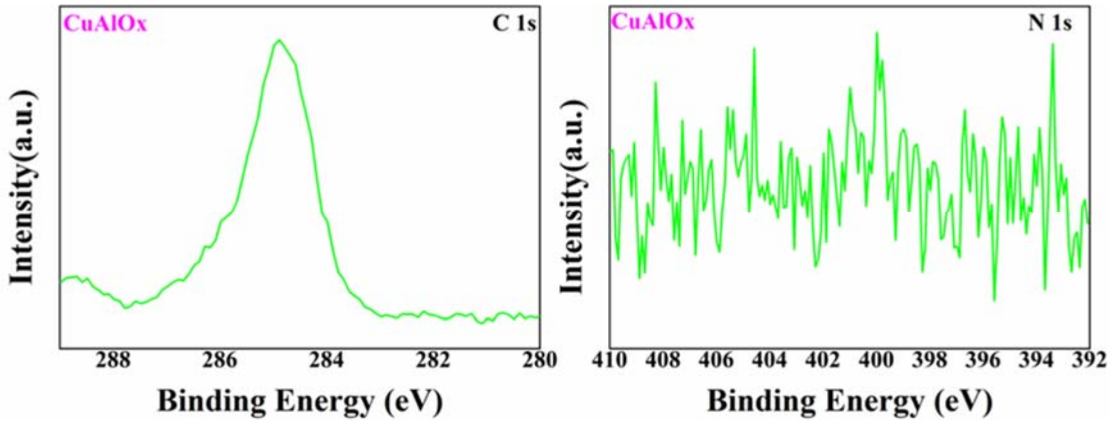

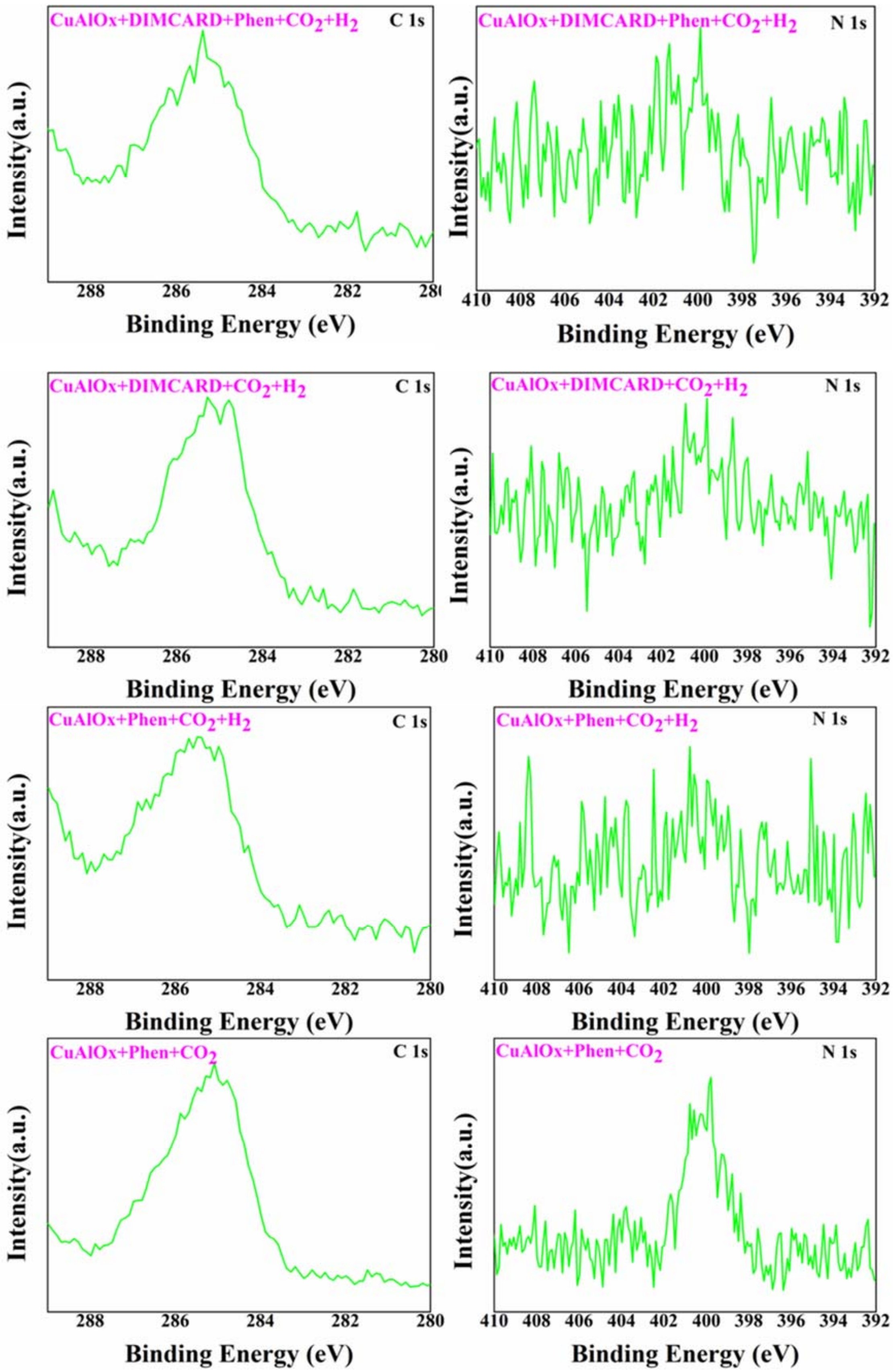
CuAlOx+DIMCARD+Phen+CO2+H2
C 1s
Intensity(a.u.)
288 286 284 282 28
Binding Energy (eV)
CuAlOx+DIMCARD+Phen+CO2+H2
N 1s
Intensity(a.u.)
410 408 406 404 402 400 398 396 394 392
Binding Energy (eV)
CuAlOx+DIMCARD+CO2+H2
C 1s
Intensity(a.u.)
288 286 284 282 280
Binding Energy (eV)
CuAlOx+DIMCARD+CO2+H2
N 1s
Intensity(a.u.)
410 408 406 404 402 400 398 396 394 392
Binding Energy (eV)
CuAlOx+Phen+CO2+H2
C 1s
Intensity(a.u.)
288 286 284 282 280
Binding Energy (eV)
CuAlOx+Phen+CO2+H2
N 1s
Intensity(a.u.)
410 408 406 404 402 400 398 396 394 392
Binding Energy (eV)
CuAlOx+Phen+CO2
C 1s
Intensity(a.u.)
288 286 284 282 280
Binding Energy (eV)
CuAlOx+Phen+CO2
N 1s
Intensity(a.u.)
410 408 406 404 402 400 398 396 394 392
Binding Energy (eV)

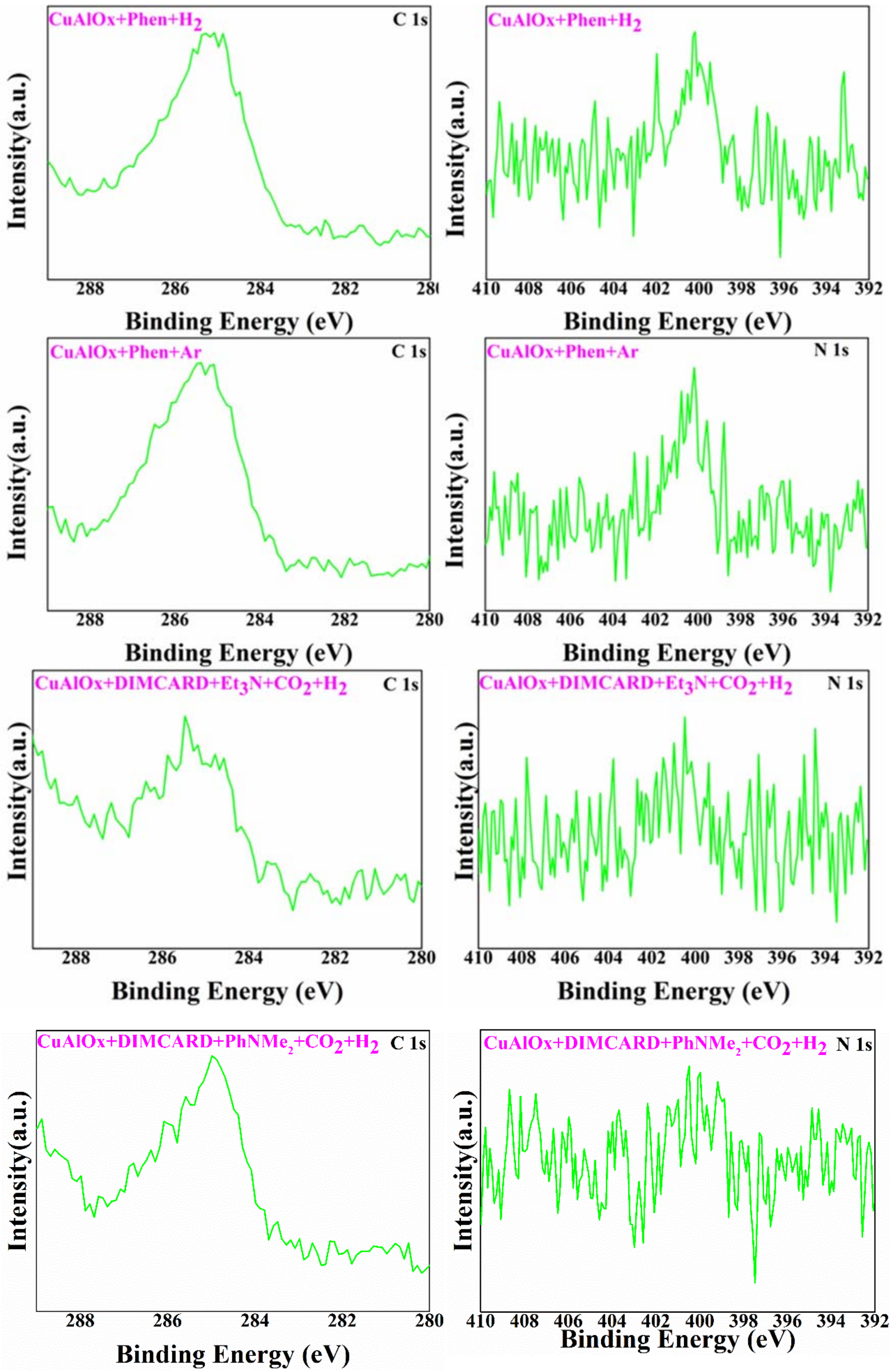

CuAlOx+Phen+H2
C 1s
Intensity(a.u.)
288 286 284 282 280
Binding Energy (eV)
CuAlOx+Phen+H2
Intensity(a.u.)
410 408 406 404 402 400 398 396 394 392
Binding Energy (eV)
CuAlOx+Phen+Ar
C 1s
Intensity(a.u.)
288 286 284 282 280
Binding Energy (eV)
CuAlOx+Phen+Ar
N 1s
Intensity(a.u.)
410 408 406 404 402 400 398 396 394 392
Binding Energy (eV)
CuAlOx+DIMCARD+Et3N+CO2+H2
C 1s
Intensity(a.u.)
288 286 284 282 280
Binding Energy (eV)
CuAlOx+DIMCARD+Et3N+CO2+H2
N 1s
Intensity(a.u.)
410 408 406 404 402 400 398 396 394 392
Binding Energy (eV)
CuAlOx+DIMCARD+PhNMe2+CO2+H2
C 1s
Intensity(a.u.)
288 286 284 282 280
Binding Energy (eV)
CuAlOx+DIMCARD+PhNMe2+CO2+H2
N 1s
Intensity(a.u.)
410 408 406 404 402 400 398 396 394 392
Binding Energy (eV)

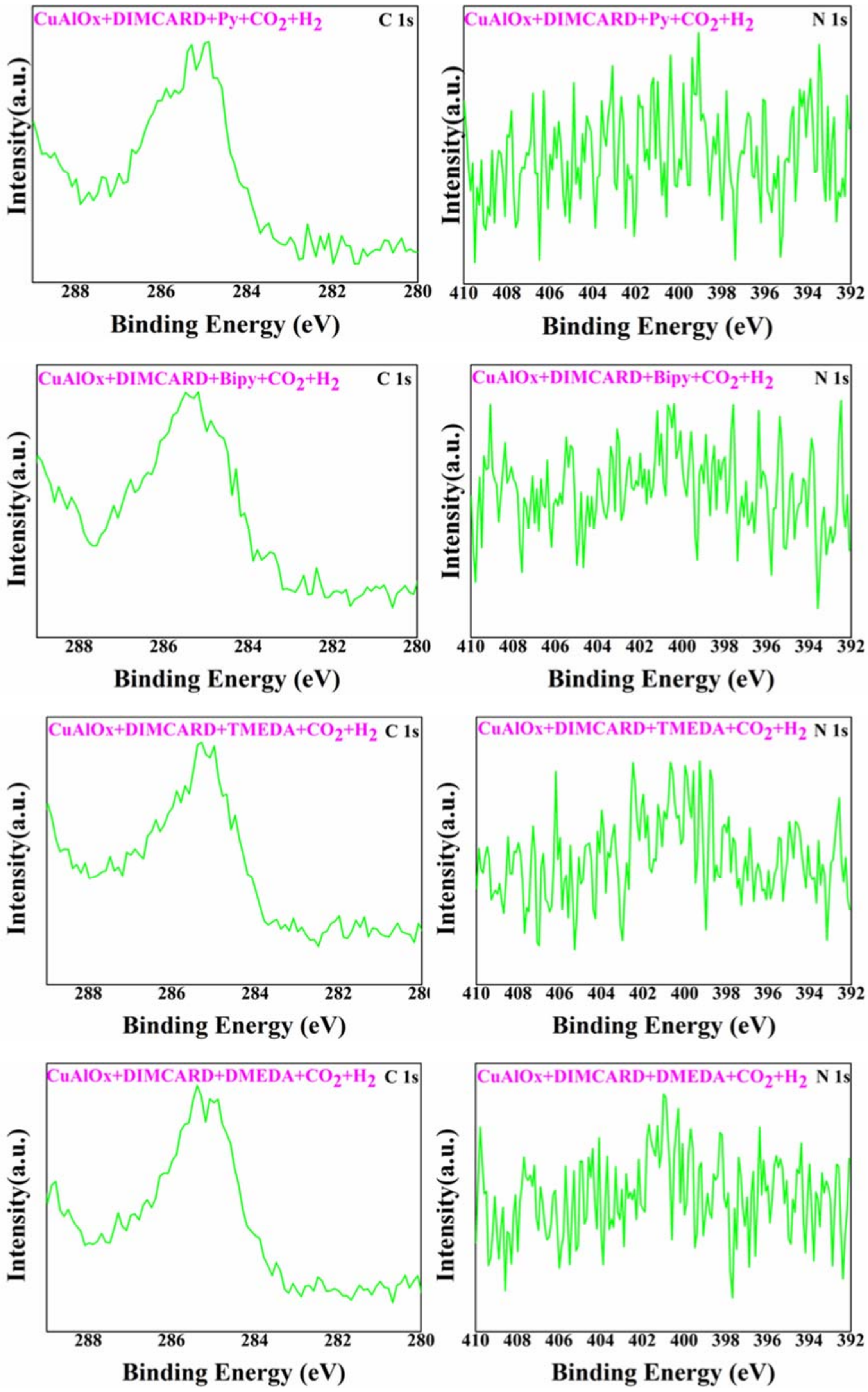

Supplementary Figure 3 | The C 1s and N 1s spectra of the catalysts.

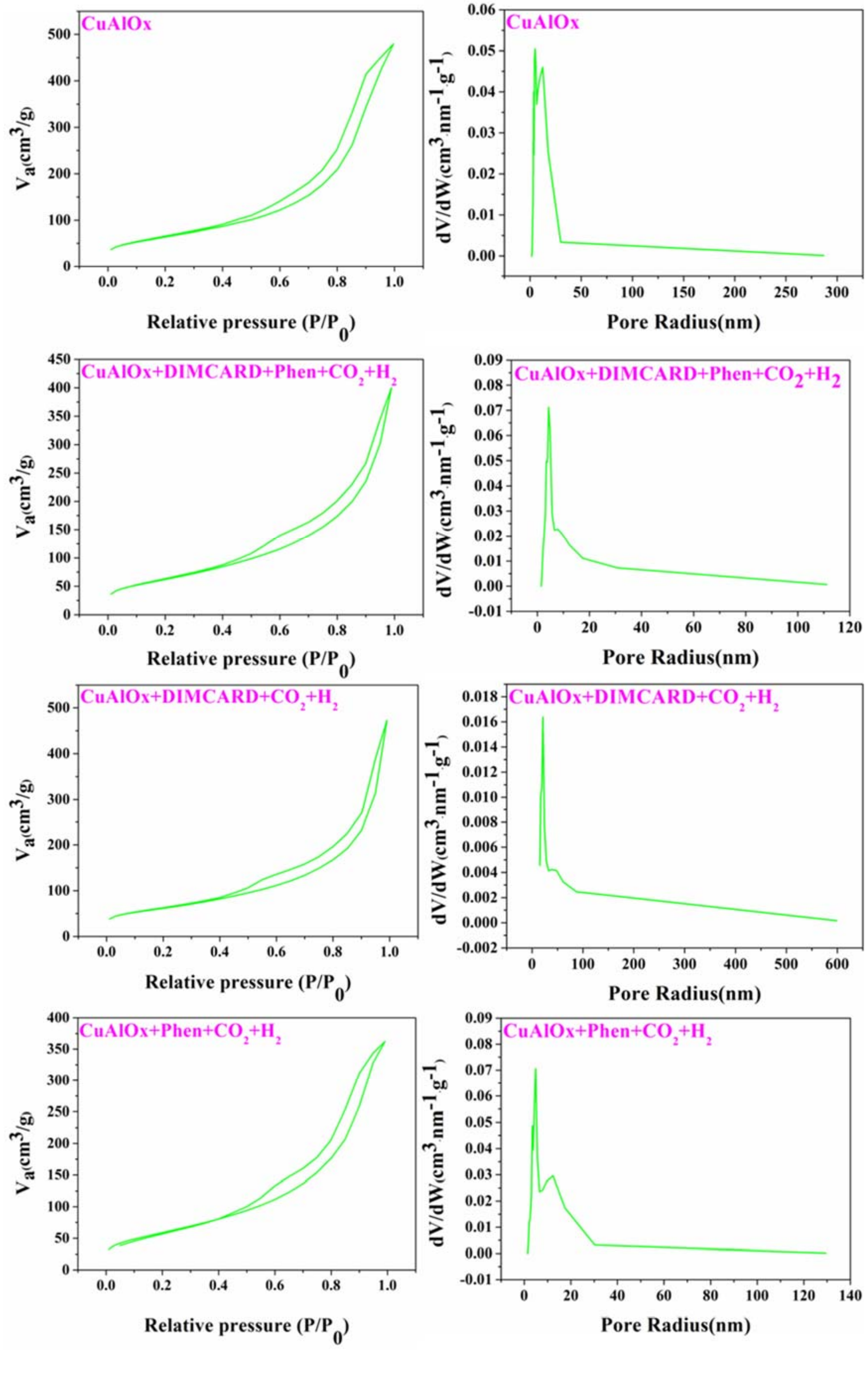
CuAlOx
Va(cm3/g)
Relative pressure (P/P0)
dV/dW(cm3.nm-1.g-1)
Pore Radius(nm)
CuAlOx+DIMCARD+Phen+CO2+H2
CuAlOx+DIMCARD+CO2+H2
CuAlOx+Phen+CO2+H2

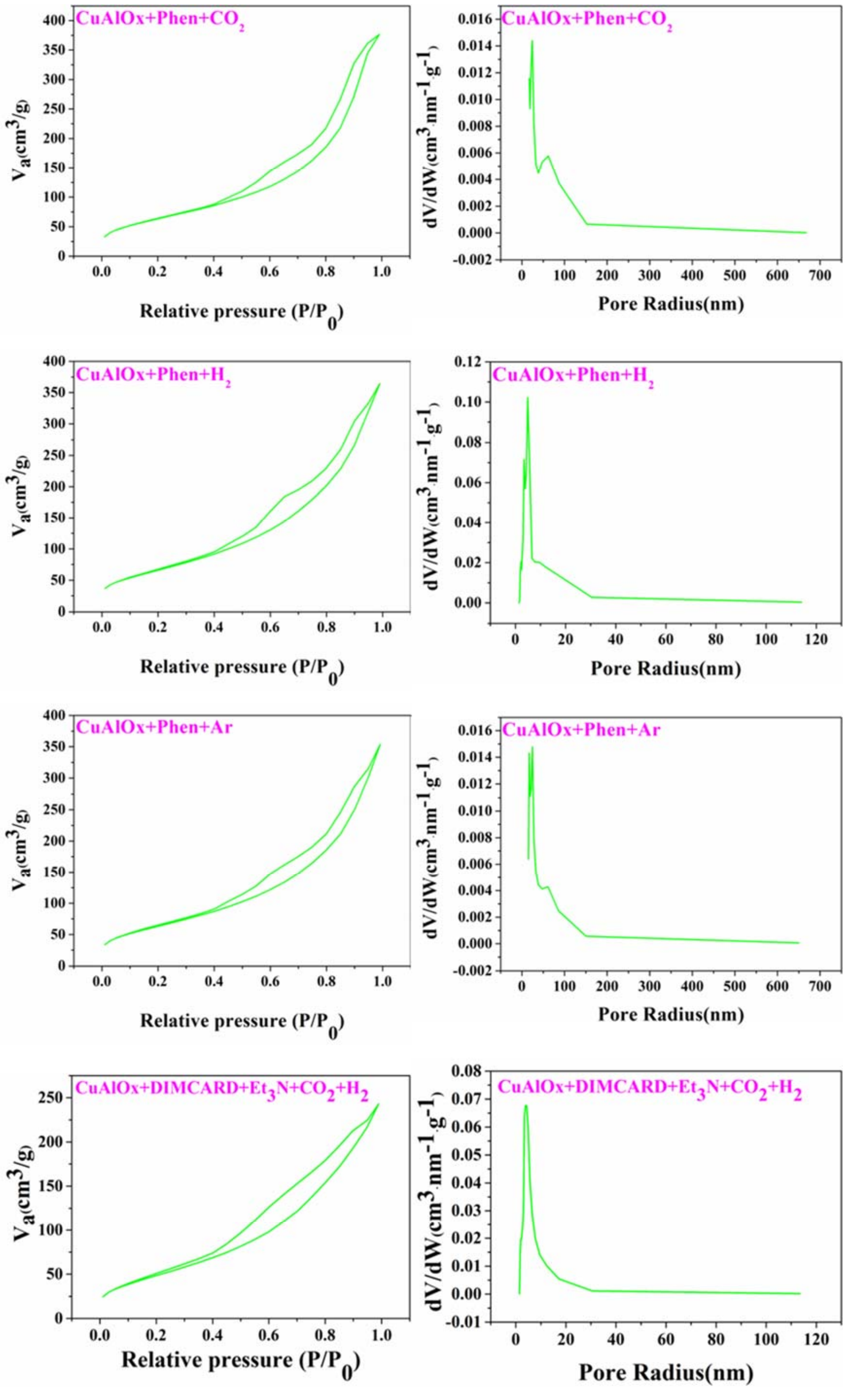

CuAlOx+Phen+CO2
Va(cm3/g)
Relative pressure (P/P0)
CuAlOx+Phen+CO2
dV/dW(cm3.nm-1.g-1)
Pore Radius(nm)
CuAlOx+Phen+H2
Va(cm3/g)
Relative pressure (P/P0)
CuAlOx+Phen+H2
dV/dW(cm3.nm-1.g-1)
Pore Radius(nm)
CuAlOx+Phen+Ar
Va(cm3/g)
Relative pressure (P/P0)
CuAlOx+Phen+Ar
dV/dW(cm3.nm-1.g-1)
Pore Radius(nm)
CuAlOx+DIMCARD+Et3N+CO2+H2
Va(cm3/g)
Relative pressure (P/P0)
CuAlOx+DIMCARD+Et3N+CO2+H2
dV/dW(cm3.nm-1.g-1)
Pore Radius(nm)

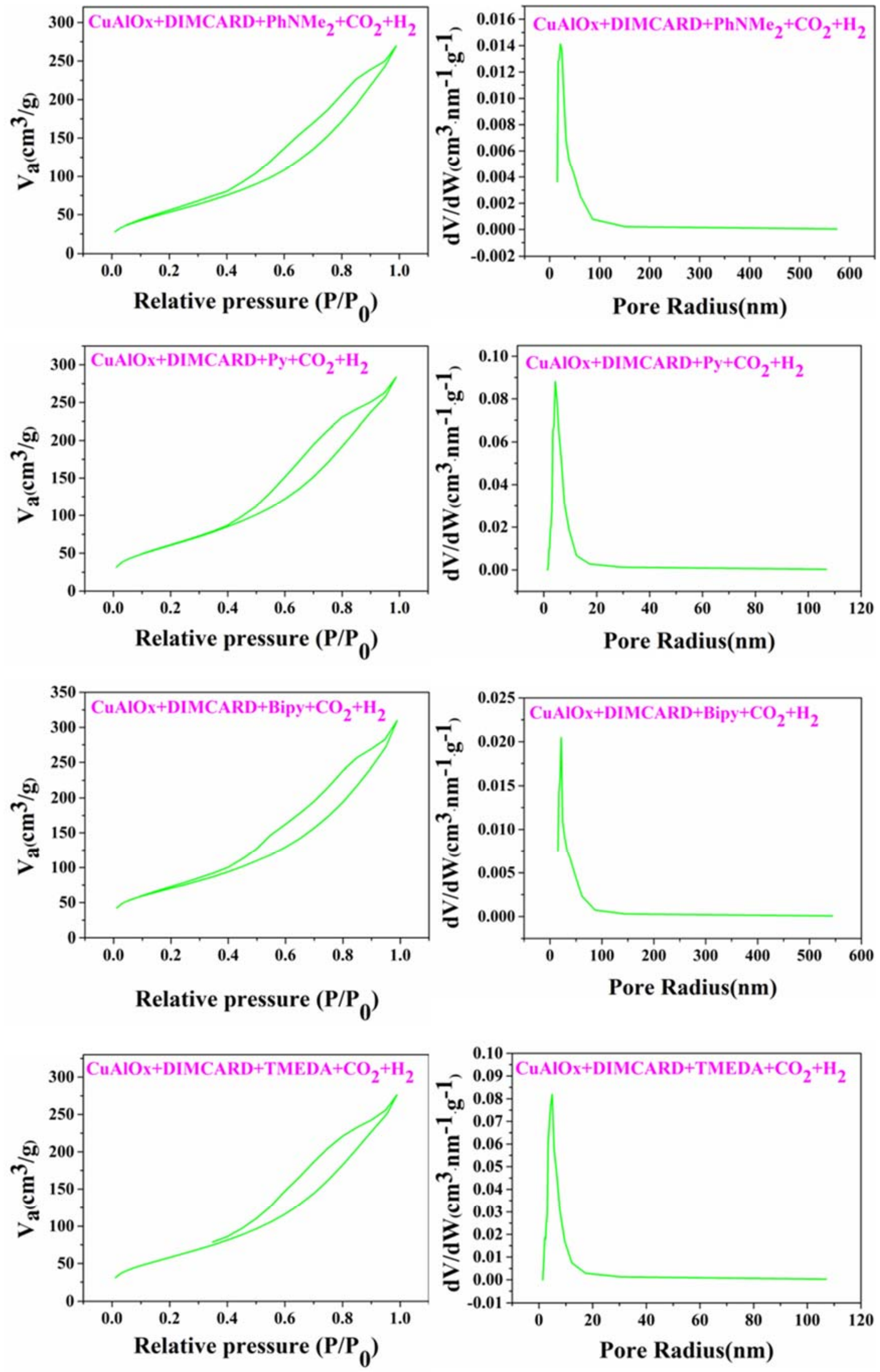
CuAlOx+DIMCARD+PhNMe2+CO2+H2
Va(cm3/g)
Relative pressure (P/P0)
dV/dW(cm3.nm-1.g-1)
Pore Radius(nm)
CuAlOx+DIMCARD+Py+CO2+H2
CuAlOx+DIMCARD+Bipy+CO2+H2
CuAlOx+DIMCARD+TMEDA+CO2+H2

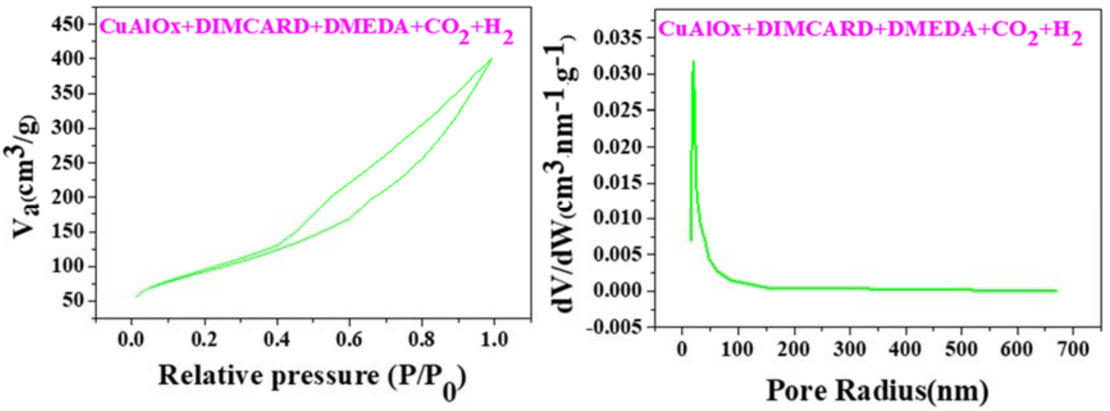

Supplementary Figure 4 | N_2 adsorption-desorption analysis of the catalysts.

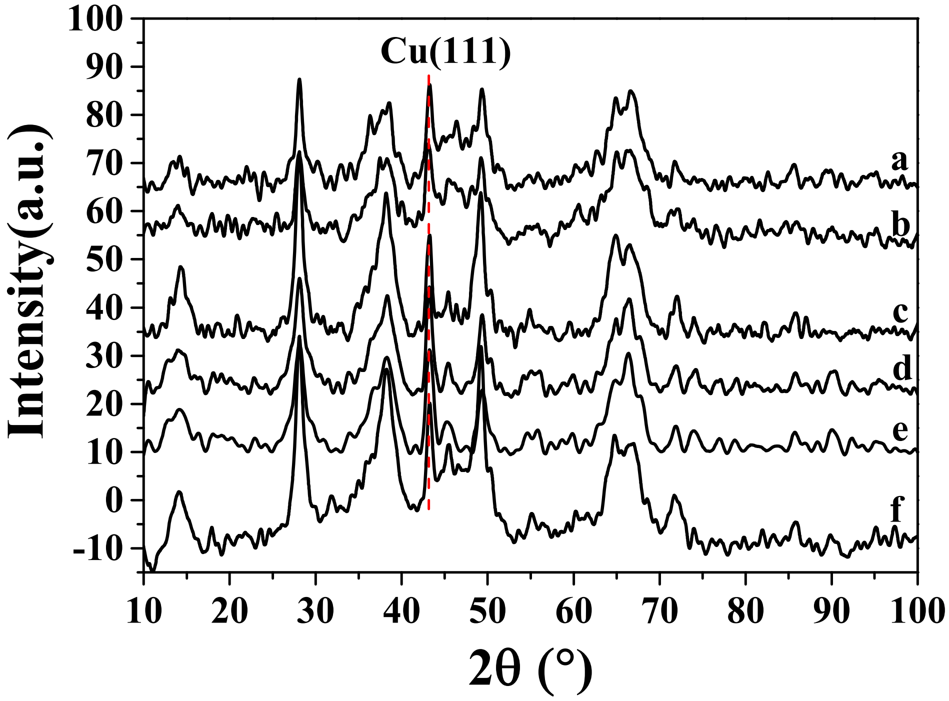

Supplementary Figure 5 | XRD patterns of treated CuAlOx catalysts. a, $CuAlO_x$+DIMCARB+Et_3N+CO_2+H_2; **b**, $CuAlO_x$+DIMCARB+$PhNMe_2$+CO_2+H_2; **c**, $CuAlO_x$+DIMCARB+Py+CO_2+H_2; **d**, $CuAlO_x$+DIMCARB+Bipy+CO_2+H_2; **e**, $CuAlO_x$+DIMCARB+TMEDA+CO_2+H_2; **f**, $CuAlO_x$+DIMCARB+DMEDA+CO_2+H_2.

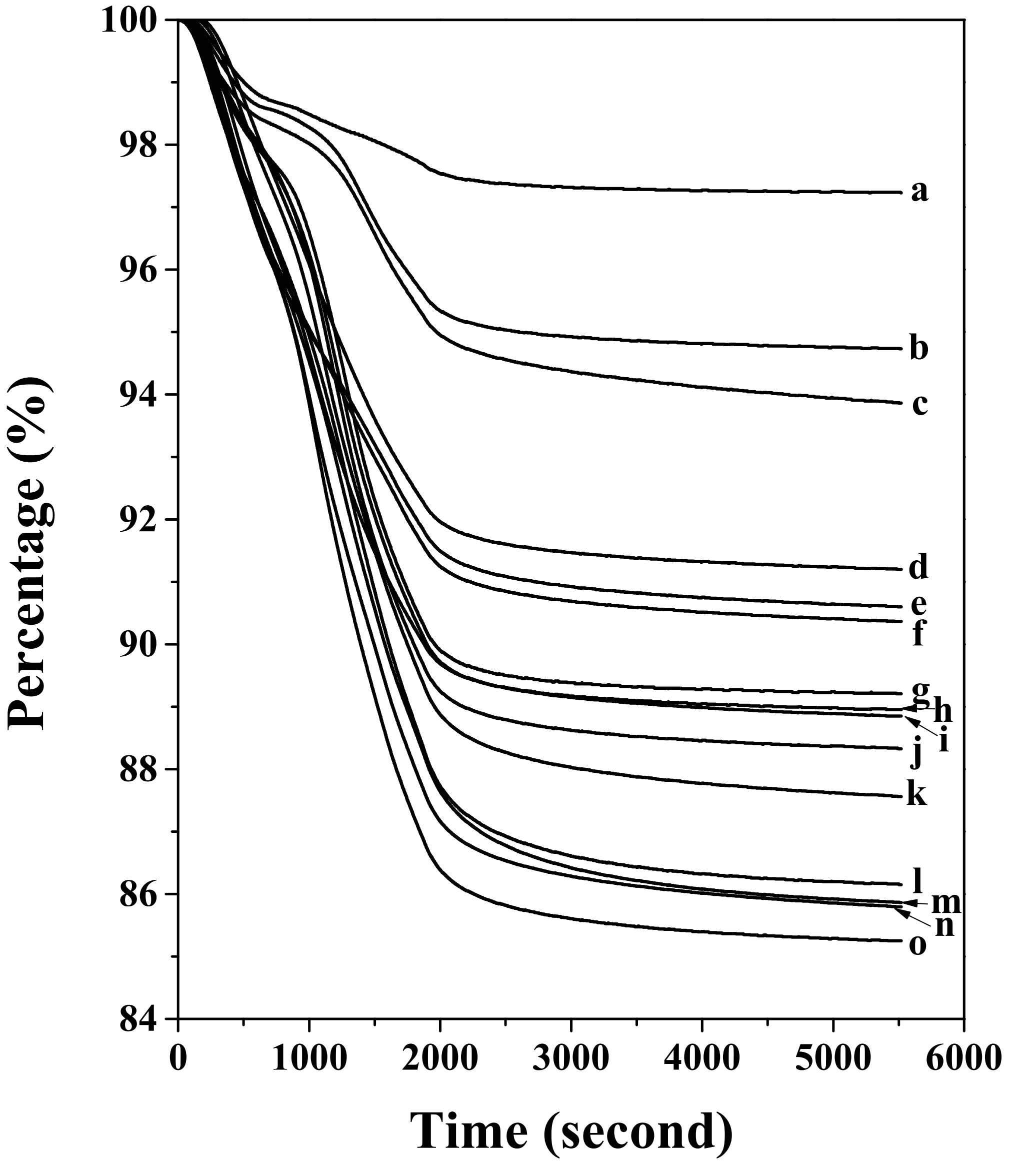

Supplementary Figure 6 | TG curves of the samples. a, $CuAlO_x$; **b,** $CuAlO_x$+Phen+H_2-8 h; **c,** $CuAlO_x$+Phen+H_2-16 h; **d,** $CuAlO_x$+Phen+H_2-24 h; **e,** $CuAlO_x$+Phen+Ar; **f,** $CuAlO_x$+Phen+CO_2; **g,** $CuAlO_x$+DIMCARB+TMEDA+CO_2+H_2; **h,** $CuAlO_x$+DIMCARB+Py+CO_2+H_2; **i,** $CuAlO_x$+Phen+CO_2+H_2; **j,** $CuAlO_x$+DIMCARB+Bipy+CO_2+H_2; **k,** $CuAlO_x$+DIMCARB+Phen+CO_2+H_2; **l,** $CuAlO_x$+DIMCARB+$PhNMe_2$+CO_2+H_2; **m,** $CuAlO_x$+DIMCARB+Et_3N+CO_2+H_2; **n,** $CuAlO_x$+DIMCARB+CO_2+H_2; **o,** $CuAlO_x$+DIMCARB+DMEDA+CO_2+H_2.

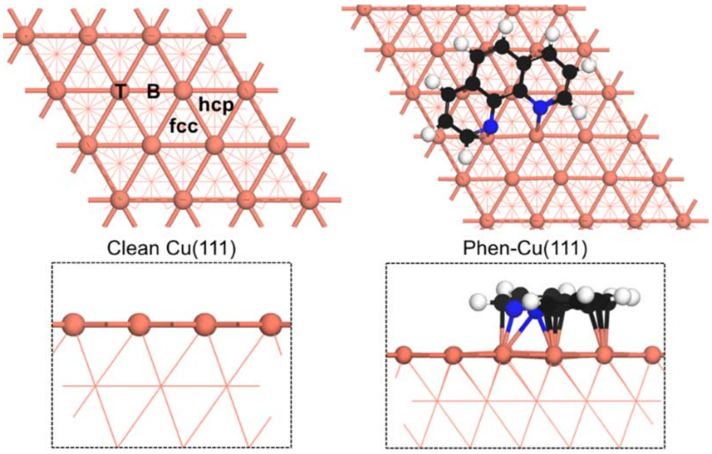

Supplementary Figure 7 | Surface models.

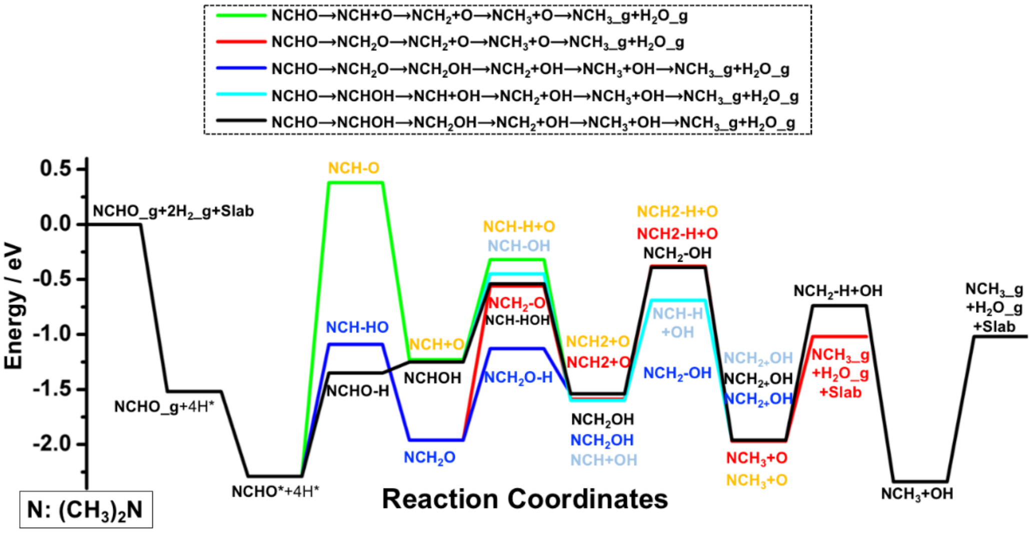

Supplementary Figure 8 | DMF hydrogenation to $N(CH_3)_3$ on clean Cu(111) surface.

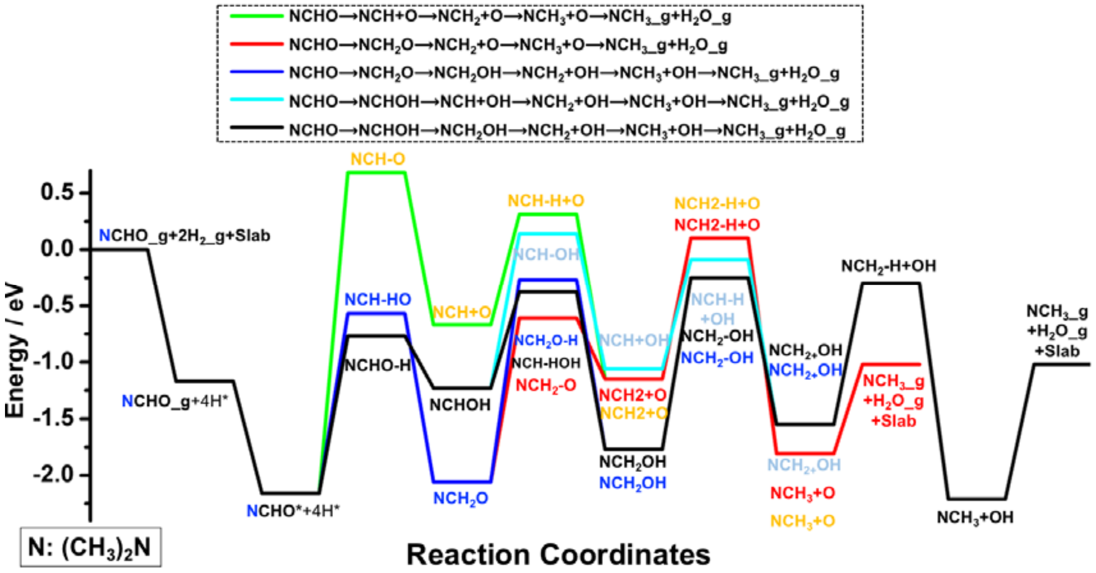

Supplementary Figure 9 | PES of DMF hydrogenation to $N(CH_3)_3$ on Phen-Cu(111).

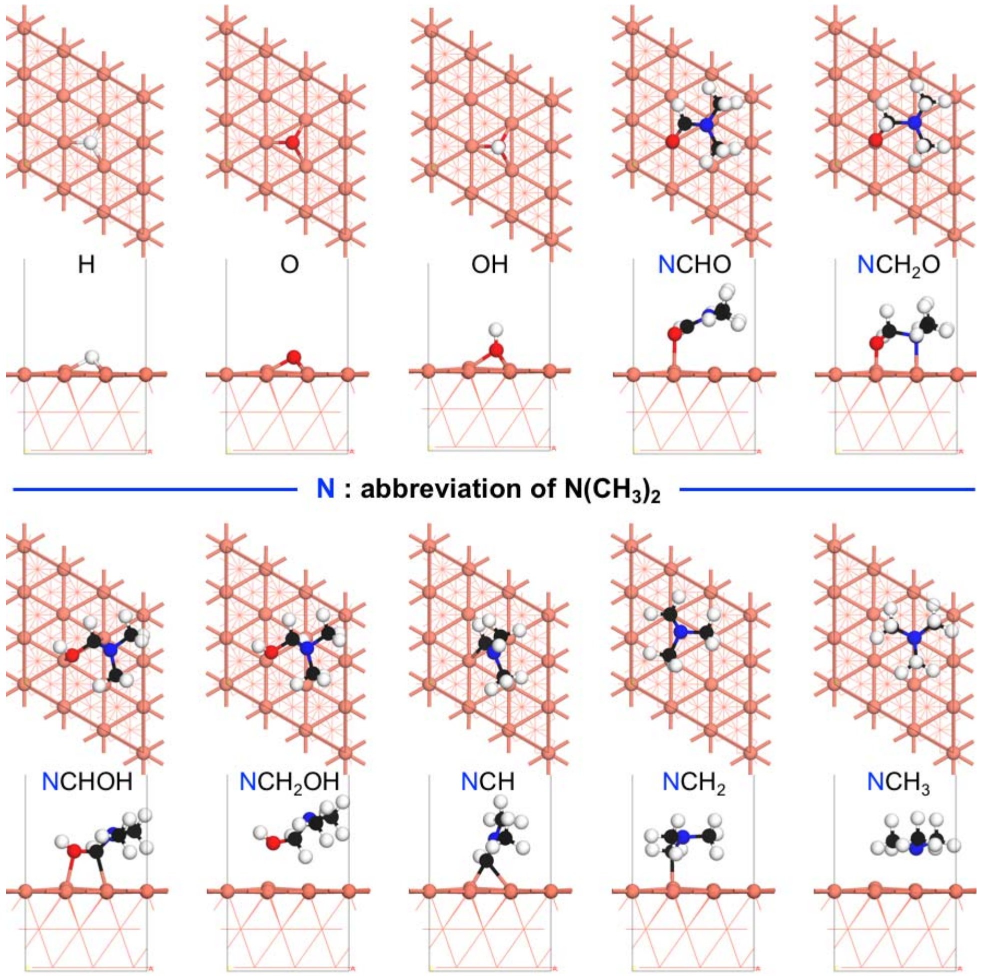

Supplementary Figure 10 | Adsorbates adsorption on clean Cu(111).

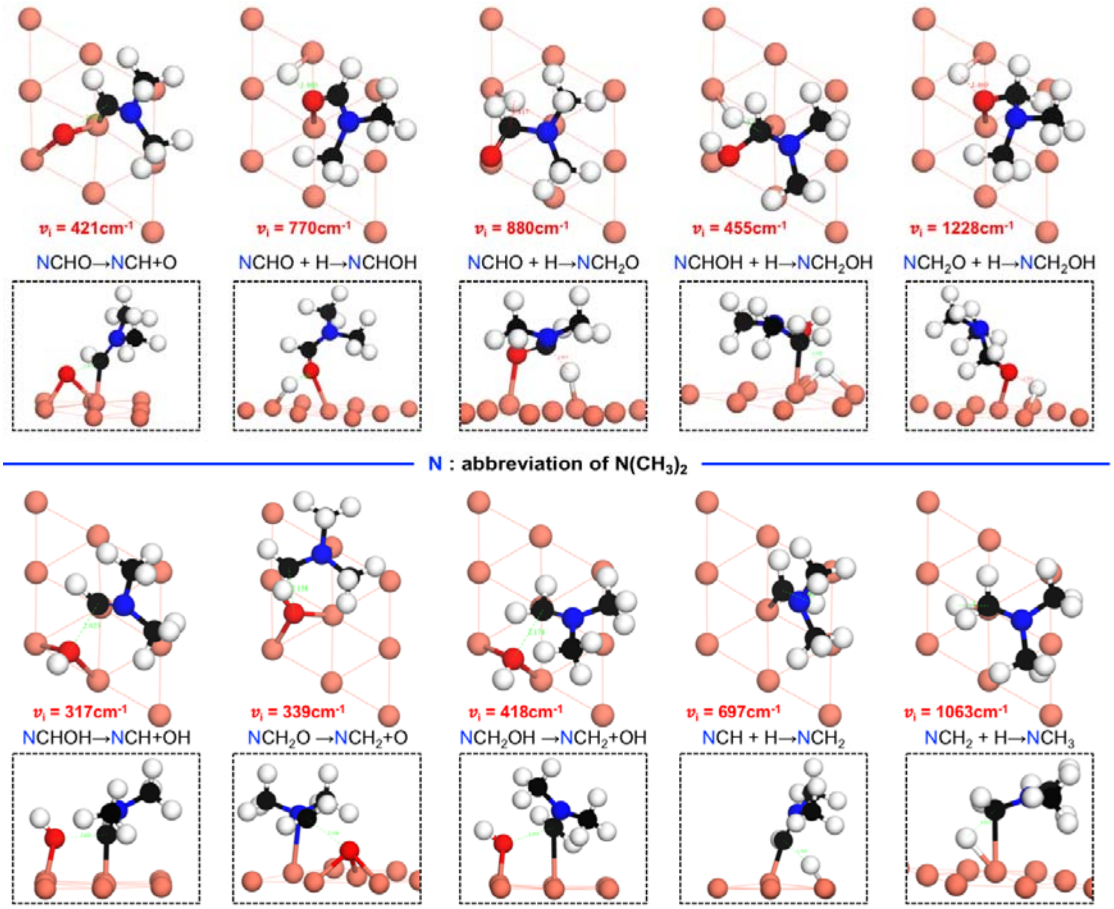

Supplementary Figure 11 | Imaginary frequencies of each TS on clean Cu(111).

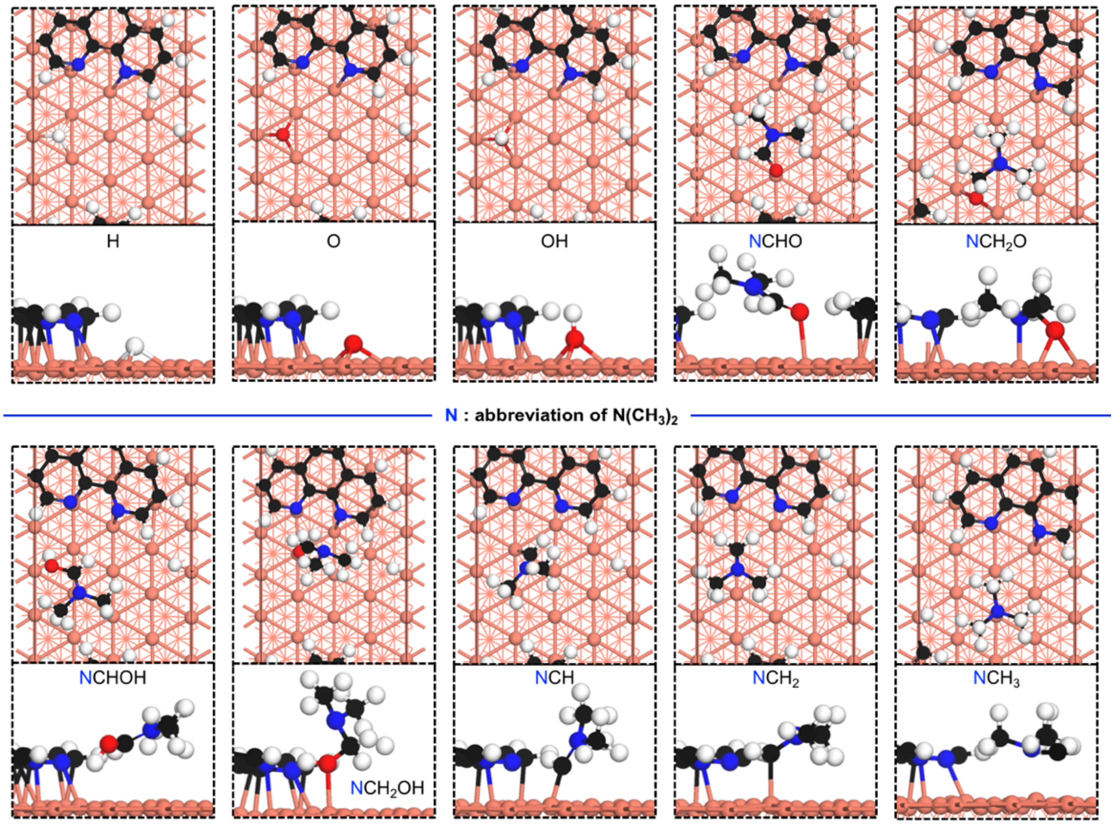

Supplementary Figure 12 | Adsorbates on phen-Cu(111).

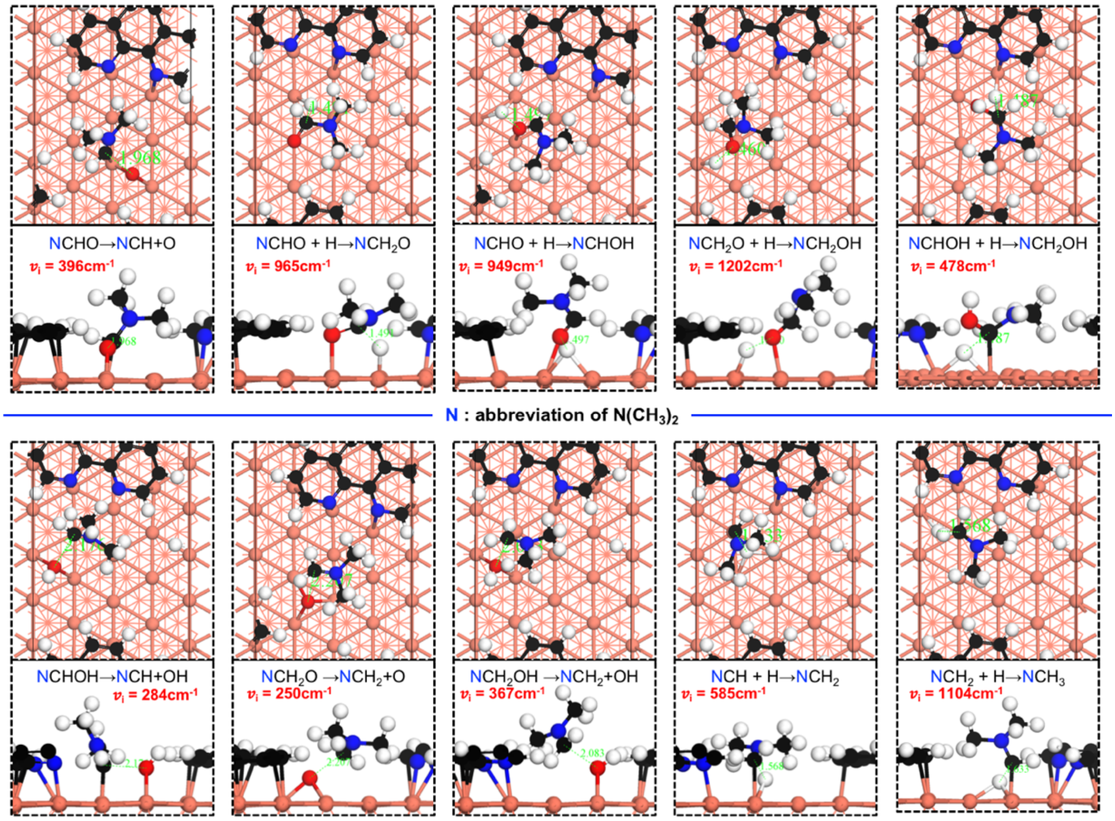

Supplementary Figure 13 | Imaginary frequencies of each TS on phen-Cu(111).

Supplementary References

1 Kresse, G. & Furthmüller, J. Efficiency of ab-initio total energy calculations for metals and semiconductors using a plane-wave basis set. *Comput. Mater. Sci.* **6,** 15-50 (1996).

2 Kresse, G. & Furthmüller, J. Efficient iterative schemes for ab initio total-energy calculations using a plane-wave basis set. *Phys. Rev. B* **54,** 11169-11186 (1996).

3 Blöchl, P. E. Projector augmented-wave method. *Phys. Rev. B* **50,** 17953-17979 (1994).

4 Kresse, G. From ultrasoft pseudopotentials to the projector augmented-wave method. *Phys. Rev. B* **59,** 1758-1775 (1999).

5 Perdew, J. P., Burke, K. & Ernzerhof, M. Generalized gradient approximation made simple. *Phys. Rev. Lett.* **77,** 3865-3868 (1996).

6 Methfessel, M. & Paxto, A. T. High-precision sampling for brillouin-zone integration in metals. *Phys. Rev. B* **40,** 3616-3621 (1989).

7 Steinmann, S. N. & Corminboeuf, C. Comprehensive benchmarking of a density-dependent dispersion correction. *J. Chem. Theory Comput.* **7,** 3567-3577 (2011).

8 Henkelman, B. P. Uberuaga, H. Jónsson, A climbing image nudged elastic band method for finding saddle points and minimum energy paths. *J. Chem. Phys.* **113**, 9901−9904 (2000).
